# Deficiency of PPP6C protects TNF-induced necroptosis through activation of TAK1

**DOI:** 10.1038/s41419-022-05076-1

**Published:** 2022-07-16

**Authors:** Yonggang Zou, Qi Zheng, Bin Jiang, Yuning Liu, Yanhua Xu, Liang Ma, Zonghao Hu, Ming Wu, Hai Song

**Affiliations:** 1 The MOE Key Laboratory of Biosystems Homeostasis & Protection, Zhejiang Provincial Key Laboratory for Cancer Molecular Cell Biology and Innovation Center for Cell Signaling Network, Life Sciences Institute, Zhejiang University, Hangzhou, Zhejiang 310058 China; 2 Department of Thoracic and Cardiovascular Surgery, The First Affiliated Hospital of Zhejiang University, Hangzhou, 310009 China; 3 Department of Thoracic Surgery, Second Affiliated Hospital, School of Medicine, Zhejiang University, Hangzhou, 310009 China

**Keywords:** Kinases, Cell signalling

## Abstract

Necroptotic cell death is mediated by a super-molecular complex called necrosome which consists of receptor-interacting protein kinase 1 and 3 (RIPK1, RIPK3) and mixed-lineage kinase domain-like protein (MLKL). The role of these kinases has been extensively investigated in the regulation of necroptosis. However, whether the protein phosphatase is involved in necroptosis is still largely unknown. Here, we identified protein phosphatase 6 catalytic subunit (PPP6C) promotes TNF-induced necroptosis by genome-wide CRISPR/Cas9 library screening. We found that *PPP6C* deficiency protects cells from TNF-induced necroptosis in a phosphatase-activity-dependent manner. Mechanistically, PPP6C acts as a TGF-β activated kinase 1 (TAK1) phosphatase to inactivate its kinase activity. Deletion of PPP6C leads to hyperactivation of TAK1 and reduced RIPK1 kinase activity upon TNF stimulation. We further showed that heterozygous deletion of *Ppp6c* in mouse gastrointestinal tract alleviates necroptosis-related tissue injury and inflammation. Thus, our study identifies PPP6C as an important regulator of necroptosis and highlights a central role of phosphatase in the regulation of necroptosis-related diseases.

## Introduction

Necroptosis, a lytic form of programmed cell death, is characterized by organelle swelling, plasma membrane rupture, and release of intracellular components that could elicit an innate immune response [1, 2]. In general, necroptosis is mediated by the receptor-interacting protein kinase 1 (RIPK1)-RIPK3-mixed-lineage kinase domain-like protein (MLKL) signaling cascade, and triggered by various stimuli, such as tumor necrosis factor (TNF), Fas, as well as TRAIL ligands [3, 4]. RIPK3 and MLKL are the crucial effectors of necroptosis, since RIPK1 is sometimes dispensable, for example, in Toll-like Receptor 3 or 4 (TLR3 or TLR4)-induced necroptosis [5, 6]. TNF is a widely-used cytokine that induces canonical necroptosis. Under TNF stimulation, tumor necrosis factor receptor 1 (TNFR1) triggers the recruitment of TNFR-associated factor 2/5 (TRAF2/5), cellular inhibitors of apoptosis (cIAPs), TNFR1-associated death domain protein (TRADD), and RIPK1 to form complex I [7, 8]. Within the complex I, RIPK1 is rapidly conjugated with ubiquitin chains which create a scaffold to recruit and activate TGF-β activated kinase 1 (TAK1)/TAK1-binding protein 2/3 (TAB2/3) complex and the inhibitor of nuclear factor-κB (NF-κB) kinase (IKK) complex (NEMO-IKKα/β), which then activates the mitogen-activated protein kinase (MAPK) and canonical NF-κB signaling pathways to prevent cell death through promoting the transcription of pro-survival genes [9]. Accordingly, when cIAPs, NEMO or TAK1 is inhibited or depleted, RIPK1 dissociates from the membrane and moves into cytoplasm to form complex II with RIPK3, Fas-associated via death domain (FADD), and caspase-8, inducing RIPK1 kinase activity-dependent apoptosis [10]. If caspase-8 is genetically depleted or inhibited by the use of Z-VAD, RIPK1 autophosphorylates and recruits RIPK3 via its homotypic RIP homology interaction motif (RHIM), causing oligomerization and autophosphorylation of RIPK3 [11–14]. Subsequently, RIPK3 phosphorylates MLKL within its C-terminal pseudokinase domain, which provokes the killing activity of MLKL by forming active oligomers that translocate to and rupture the plasma membrane [12, 15–19].

TAK1 is considered as a central regulator of cell death and has been demonstrated to play a pro-survival role via the NF-κB and MAPK activation [20, 21]. Recent studies unveiled that TAK1 also regulates necroptosis independent of its role in NF-κB activation [10, 22, 23]. Previous studies showed that TAK1 directly or indirectly via P38 and MAPK activated protein kinase 2 (MAPKAPK2, also known as MK2) axis phosphorylates RIPK1 at S320/321 to inhibit RIPK1 activation [24–26]. IKKα/β were also reported to inhibit necroptosis by regulating RIPK1 phosphorylation at S25 [27]. In addition, cells are more sensitive to RIPK1-dependent apoptosis upon TNF stimulation in the absence of functional TAK1 [23]. Thus, TAK1 plays an anti-necroptosis role from diverse perspectives. However, one research found that deletion of *Tab2* in mouse embryonic fibroblast (MEF) cells results in sustained activation of TAK1, but unexpectedly aggravates necroptosis following TNF stimulation [28]. These studies suggest that TAK1 plays multiple roles in the determination of cell fate upon TNF treatment.

Protein phosphatase 6 (PP6), a member of the phosphoprotein phosphatase (PPP) family of Ser/Thr protein phosphatases, is a trimetric holoenzyme, consisting of catalytic, structural, and regulatory subunits with the catalytic subunit designated PPP6C [29]. PPP6C is highly conserved throughout eukaryotes including human, and exhibits multiple roles in regulating several cellular processes. PPP6C was previously identified as a negative regulator of NF-κB signaling by stabilizing NF-κB inhibitor ε (IκBε) and dephosphorylating TAK1 [30, 31]. In this study, we identified PPP6C as a positive regulator of necroptosis through loss-of-function screening by utilizing a pooled genome-scale CRISPR/Cas9 library in L929 cells. We found that depletion of *Ppp6c* enhances TAK1-IKKα/β axis activity which promotes phosphorylation of RIPK1 at S25, and results in the inhibition of RIPK1 kinase activity and preventing TNF-mediated RIPK1-dependent necroptosis.

## Materials and methods

### CRISPR/Cas9 knockout library screening

We used the mouse GeCKOv2 CRISPR knockout pooled library to identify genes that regulate TNF-induced necroptisis in L929 cells. The library contains 130,209 unique sgRNA sequences targeting 20,611 mouse genes with six guides per gene (Addgene #1000000053). L929 cells were transduced with the lentivirus library at <0.3 multiplicity of infection to barcode individual cells. Puromycin was added at a final concentration of 6 μg/ml in the cell culture medium 24 h post infection for 5 days to generate a mutant cell pool. The cells were then treated with TZ (10 ng/ml TNF + 20 µM Z-VAD-FMK) for 8 h to deplete non-resistant cells. After 6-round TZ selection, at least 3 × 10^7^ cells were used for genomic DNA extraction. sgRNA sequences were amplified with forward primer GTAGAAAGTA ATAATTTCTTGGGTAGTTTGCAG and reverse primer CACTCCTTTCAAGACCTAGCTAGCGAATTCA. Then, the amplified DNA fragments were sequenced on an Illumina Hiseq.

### Cell culture and cell lines

HEK293T and MEF cells were cultured in Dulbecco’s modified Eagle medium (DMEM) (Gibco) supplemented with 10% fetal bovine serum (FBS), 100 U/ml penicillin/streptomycin. L929 and HT29 cells were maintained in Roswell Park Memorial Institute (RPMI) 1640 supplemented with 10% FBS, 100 U/ml penicillin/streptomycin. All cells were cultured under 5% CO_2_ at 37 °C. To generate *Ppp6c* knockout cells, L929 cells were infected with lentiCRISPRv2-sg*Ppp6c* lentivirus and single clone was picked. To generate TAK1 knockout cells, HEK293T were infected with lentiCRISPRv2-sgTAK1 lentivirus and single clone was picked. To generate HA-PPP6C or HA-PPP6C-PD expressing cells, L929 or *Ppp6c*-KO L929 cells were infected with pCDH-HA-PPP6C or pCDH-HA-PPP6C-PD lentivirus. *PPP6C*-knockdown HT29 cells and *Tab2*-knockdown L929 cells were generated by shRNA. Cells were infected with pLKO.1-sh*PPP6C* or pLKO.1-sh*Tab2* lentivirus and selected for puromycin-resistant cells. Guide RNA and shRNA sequences are listed in Table S1. *Ppp6c*^flox/flox^ MEFs were transduced with SV40 large T antigen-expressing lentivirus for immortalization and then with Cre-expressing lentivirus to generate *Ppp6c* KO MEFs. For transfection, plasmid DNA was transfected into HEK293T cells by Polyethylenimine (PEI). For lentivirus infection, viruses generated in HEK293T with indicated lentivirus plasmids and packaging plasmids psPAX2 (Addgene, #12260), pMD2.G (Addgene, #12259) were added to cell culture medium with 10 μg/ml Polybrene (Beyotime, C0351).

### Luciferase reporter assay

Luciferase reporter assay was performed using a dual-luciferase reporter assay system. Plasmids encoding NF-κB-driven firefly luciferase reporter and CMV promoter-driven renilla luciferase were co-transfected with FLAG-PPP6C into HEK293T cells for 24 h. Cells were treated with TNF (10 ng/ml) for 6 h, and then cell lysis was subjected to luciferase activity measurement as described in dual-luciferase reporter assay kit (Promega, USA).

### Immunoblotting and immunoprecipitation

Cells were collected in ice-cold lysis buffer (20 mM Tris-HCl, pH 7.5, 150 mM NaCl, 1 mM EDTA, 1 mM EGTA, 1% TritonX-100, 2.5 mM sodium pyrophosphate, 1 mM β-glycerophosphate, 1 mM Na_3_VO_4_ and protease inhibitor cocktail), and then centrifuged at 150,00 × *g* for 20 min at 4 °C. The supernatants were collected for immunoblotting or immunoprecipitation. For immunoprecipitation, 10 μl anti-FLAG or anti-HA magnetic beads were added to the supernatants and incubated at 4 °C for 6 h under gentle agitation. Then the beads were washed by washing buffer (20 mM Tris-HCl, pH 7.5, 150 mM NaCl, 1 mM EDTA, 1 mM EGTA, 0.5% TritonX-100, 2.5 mM sodium pyrophosphate, 1 mM β-glycerophosphate, 1 mM Na_3_VO_4_ and protease inhibitor cocktail) for three times. Finally, the beads were mixed with SDS loading buffer and the eluted protein was used for immunoblotting. Antibody information is listed in Table S2.

### RNA extraction and quantitative RT-PCR

Total RNA was isolated from indicated cells using the TRIzol reagent (Invitrogen, USA), according to the manufacture’s protocol. One microgram total RNA was used for cDNA synthesis using The PrimeScript™ RT reagent Kit with genomic DNA Eraser (Takara). Quantitative PCR was then performed using Hieff® qPCR SYBR Green Master Mix (Yeasen). Sequences of the primers are listed in Table S1.

### Cell death and survival assay

For L929 cells, necroptosis was induced by the treatment with TZ (10 ng/ml mouse TNF + 20 μM Z-VAD-FMK). For HT29 cells, necroptosis was induced by TSZ treatment, which is a combination of 20 ng/ml TNF, 0.2 μM SM-164, and 20 μM Z-VAD-FMK. To measure the cell death after treatment, PI (propidium iodide) was directly added in the medium at a concentration of 5 μg/ml and incubated for 15 min. The visual cell death was photographed by an inverted fluorescence microscope and the percentage of PI incorporation was quantified by flow cytometer (Beckman CytoFlex S). Cell viability was measured by Cell Counting Kit 8 (CCK8).

### Animal work

*Ppp6c* conditional knockout mice were a gift from Dr. Xingzhi Xu. *Ppp6c*^flox/flox^ mice were bred to *Shh*^*Cre/+*^ mice to generate *Shh*^*Cre/+*^;*Ppp6c*^f/+^ mice. All mice were bred on a C57BL/6J genetic background. *Shh*^*Cre/+*^;*Ppp6c*^f/+^ mice and their littermate controls *Ppp6c*^flox/+^ mice were bred by crossing *Shh*^*Cre/+*^ with *Ppp6c*^f/f^. For systemic inflammatory response syndrome (SIRS) mouse model, male mice aged at 6–8 weeks were injected intravenously through tail vein with mouse TNF diluted in endotoxin-free PBS (0.25 μg/g per mouse). Control mice were injected with PBS. For DSS-induced colitis, male mice aged at 6–8 weeks were supplied with dextran sulfate sodium (DSS) in drinking water 3% (w/v) for 7 days followed by regular water for 1 day. DSS water was replaced on day 2 and day 4. Body weight was measured daily for up to 8 days, and the mice were then sacrificed to enable colon length measurement, histology analysis, and colonic proteins extraction for immunoblotting analysis. All mice were housed in specific pathogen-free condition with 12-h light/dark cycle and freely access to food and water at the Zhejiang University Laboratory Animal Center. All mouse experiments were approved by the Institutional Animal Care and Use Committee of Zhejiang University.

### Histology

Mouse colons were fixed in 4% paraformaldehyde for 6 h. The fixed tissues were dehydrated in gradient ethanol, cleared in xylene, and embedded in paraffin. Five-micrometer sections were cut and mounted on adhesion microscope slides. Sections were then used for hematoxylin and eosin (H&E) or terminal dexynucleotidyl transferase (TdT)-mediated dUTP nick end labeling (TUNLE) staining using TUNEL Apoptosis Detection Kit (FITC) (Yeasen, Shanghai, China) according to the manufacturer’s instruction.

### Immunofluorescence

L929 cells were plated on coverslips and treated with TNF (10 ng/ml) for the indicated time, and then fixed with pre-cooled methanol on ice for 10 min, followed by blocking with 3% BSA at room temperature for 30 min. Cells were incubated with primary antibodies diluted in 3% BSA at 1:500 for 2 h at room temperature. After washed for three times with PBST, cells were incubated with secondary antibodies in 3% BSA for 2 h. Nuclei were stained with 0.5 μg/ml of DAPI at room temperature for 10 min. Images were acquired on a confocal microscope.

### Production of recombinant HA-mTNF proteins

N-terminal HA-tagged mouse TNF (mTNF) construct was obtained by cloning truncated mTNF cDNA (encoding Asp89-Leu235) into the pcDNA3 vector with an Igκ leader sequence and a HA tag at the N-terminus of mTNF. Conditional medium with recombinant HA-mTNF proteins was obtained by transfecting this construct into HEK293T cells for 60 h. Concentration of the recombinant HA-mTNF protein in the conditional medium was determined using commercial mTNF protein.

### Statistical analysis

Statistical analysis was performed using GraphPad Prism software (version 8.0.1; GraphPad Software). Statistical analysis was performed using an unpaired Student’s *t* test (two groups) or one-way ANOVA analysis (multiple groups). A difference was considered significant if *P* < 0.05 and statistical significance was defined as *P* < 0.05 (*), *P* < 0.01(**), and *P* < 0.001(***).

## Results

### Genome-wide CRISPR/Cas9 library screening identified PPP6C as a positive regulator of necroptosis

**Fig. 1:**
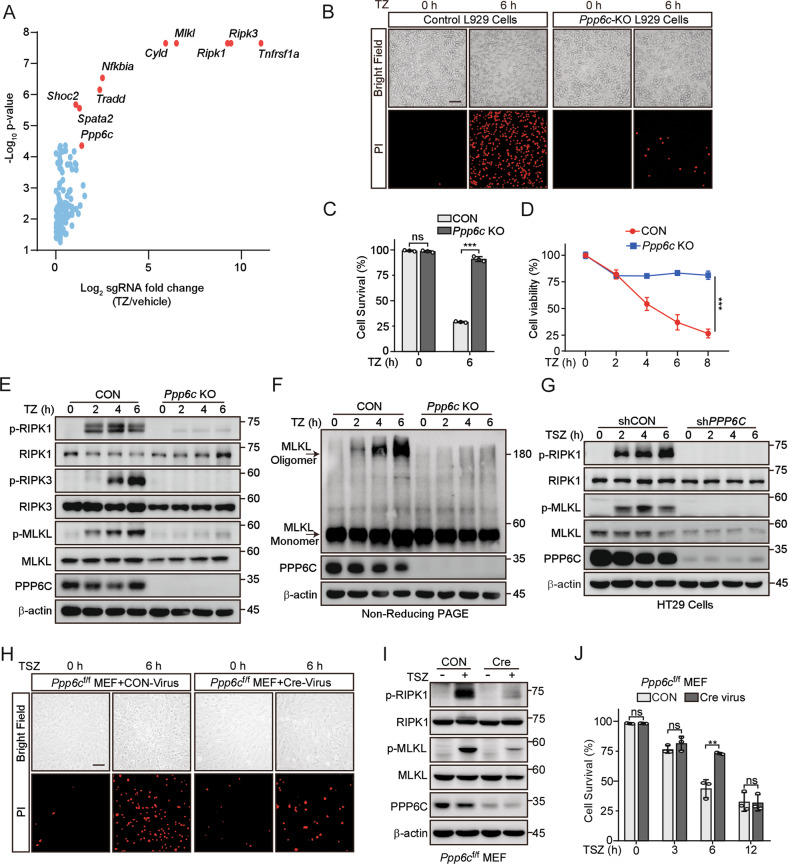
Deletion of *Ppp6c* prevents TNF-induced necroptosis. **A** Scatter diagram revealed that sgRNAs targeting *Ppp6c* were selected during TNF + Z-VAD-FMK (TZ) treatment in L929 cells. **B**, **C** Control and *Ppp6c*-KO L929 cells were treated with TZ for 6 h and analyzed by propidium iodide (PI) staining under a microscope (**B**) or quantified by flow cytometer (**C**). Scale bar, 50 μm. **D** Control and *Ppp6c*-KO L929 cells were treated with TZ for the indicated time and the cell viability was measured by CCK8. **E** Control and *Ppp6c*-KO L929 cells were treated with TZ for the indicated time and the activation of RIPK1, RIPK3 and MLKL proteins was monitored by immunoblot. **F** Control and *Ppp6c*-KO L929 cells were treated with TZ for the indicated time and cell lysates were resolved on non-reducing PAGE for immunoblot. **G** Control and *PPP6C*-knockdown HT29 cells were treated with TSZ for the indicated time and cell lysates were probed with indicated antibodies. **H**–**J** Immortalized *Ppp6c*^f/f^ MEF cells were infected with control lentivirus or Cre-Lentivirus for 72 h and followed by the treatment with TSZ for 6 h. Cells were stained with PI and analyzed under a microscope (**H**) or lysed for immunoblot with indicated antibodies (**I**), or quantified by flow cytometer after PI staining (**J**). Scale bar, 50 μm. Data shown are representative of three independent experiments and presented as means ± SDs of triplicates (**C**, **D**, **J**). **p < 0.01, ***p < 0.001, with an unpaired Student’s *t*-test (**C**, **D**, **J**).

To identify uncharacterized regulators of necroptosis, we conducted a positive-selection screening to enrich the cells acquired TNF + Z-VAD (TZ) resistance in L929 cells, which exhibit high sensitivity to TNF-induced necroptosis and are widely used in TNF-induced necroptosis study [32], by utilizing a pooled genome-scale CRISPR/Cas9 knockout (GeCKO) library. After 6 rounds of TZ selection, the sgRNA abundance in the enriched cells was determined by next-generation sequencing (NGS). The enrichment of sgRNAs was calculated and plotted (Fig. 1A). We identified a subset of sgRNAs targeting 79 genes significantly enriched (*P* < 0.05) in the TZ-treated cells when compared to vehicle control (Table S3). Among these sgRNAs, we observed the highest level of sgRNA enrichment targeting genes such as *Tnfrsf1a*, *Ripk1*, *Ripk3*, *Mlkl*, *Tradd*, *Cyld*, *Spata2* (Fig. 1A), which have been previously reported as the regulators of necroptosis [7, 33–37]. The enrichment of these genes indicates that our screening was effective.

In this screening, *Ppp6c*, the catalytic subunit of protein phosphatase 6 [38], was identified as a positively selected gene upon TZ treatment since 5 sgRNAs targeting *Ppp6c* were enriched in TZ-treated cells (Fig. S1A), implying that loss of *Ppp6c* protected L929 cells from TZ-induced necroptosis. To investigate the function of *Ppp6c* in necroptosis, we generated *Ppp6c* knockout (KO) L929 cell subclones using CRISPR/Cas9. Deletion of *Ppp6c* in L929 cells did no significantly affect the cell proliferation (Fig. S1B). Then, we treated *Ppp6c*-KO L929 cells and control cells with TZ for 6 hours and then stained with PI which is usually used to evaluate plasma membrane integrity. Consistent with the result of CRISPR/Cas9 screening, we found that *Ppp6c*-KO L929 cells were resistant to TZ-induced necroptosis (Fig. 1B, C) and displayed a higher cell viability under TZ treatment (Fig. 1D). It is well established that necroptosis signaling pathway is tightly regulated by a kinase cascade RIPK1-RIPK3 to phosphorylate MLKL [12–14, 19, 39]. To determine whether PPP6C signals through this cascade, we treated *Ppp6c*-KO and control L929 cells with TZ for 2, 4, and 6 h, and then lysed cells for western blot analysis. We found that the phosphorylation level of RIPK1 (S166) [14], RIPK3 (S232) [13], and MLKL (S345) [19] in *Ppp6c*-KO L929 cells was remarkably reduced compared with the control cells (Fig. 1E). Consistently, MLKL oligomerization was greatly inhibited upon the removal of *Ppp6c* in L929 cells (Fig. 1F). To evaluate whether these findings were conserved in human cells, we generated *PPP6C*-knockdown human HT29 cells using two independent shRNAs. Consistently, shRNA-mediated knockdown of *PPP6C* conferred HT29 cells to the resistance to TSZ-induced necroptosis by analyzing plasma membrane integrity (Fig. S1C, D). In addition, *PPP6C*-knockdown HT29 cells exhibited a higher cell viability (Fig. S1E), reduced phosphorylation level of RIPK1 (S166), and MLKL (S358, corresponding to mouse S345) [12] (Fig. 1G), and less MLKL oligomerization upon TSZ treatment (Fig. S1F). Furthermore, we isolated MEF cells from *Ppp6c*^flox/flox^ mouse embryos and infected the immortalized *Ppp6c*^flox/flox^ MEF cells with control or Cre-lentivirus to generate *Ppp6c*-KO MEF cells. Consistent with previous report [40], *Ppp6c* deletion reduced cell proliferation in MEF cells (Fig. S1G). Notably, *Ppp6c* deficiency protected MEF cells from TSZ-induced necroptosis during the early stage of the treatment (Fig. 1H, I). However, longtime exposure to TSZ eventually resulted in the cell death of *Ppp6c*-KO MEF cells (Fig. 1J), while *Ppp6c* deletion provided a long-term protection in L929 cells treated with TZ (Fig. S1H). Taken together, these data suggested that *Ppp6c* deficiency inhibits RIPK1-RIPK3-MLKL cascade-mediated necroptotic cell death.

### Phosphatase activity of PPP6C is required for its role in regulating necroptosis

**Fig. 2:**
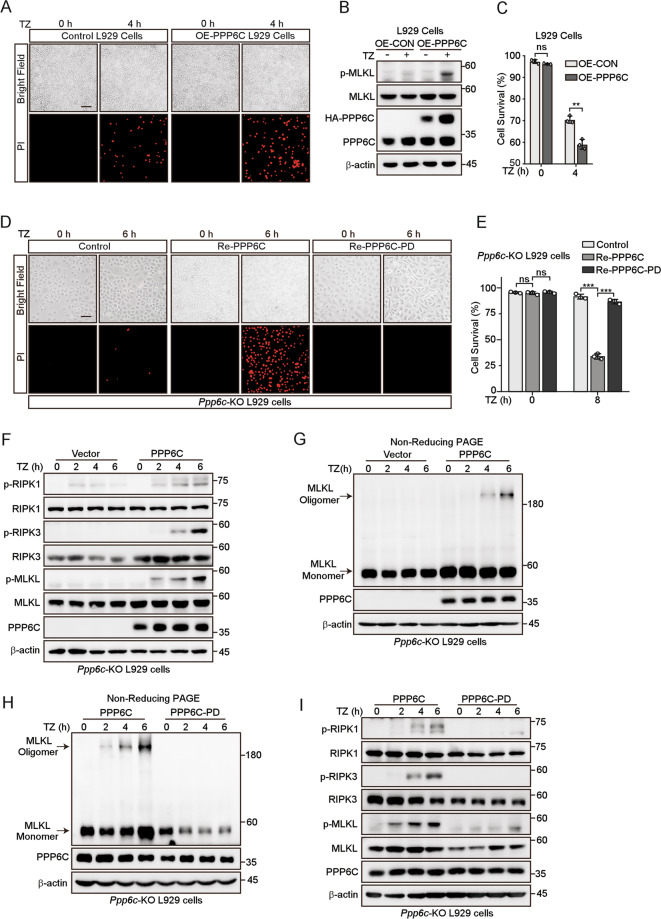
Phosphatase activity of PPP6C is required for its role in regulating necroptosis. **A**, **C** Control and PPP6C-overexpression L929 cells were treated with TZ for 4 h, then stained with PI and analyzed under a microscope (**A**), or quantified by flow cytometer (**C**). Scale bar, 50 μm. **B** Control and PPP6C-overexpression L929 cells were treated with TZ for 4 h and cell lysates were probed with indicated antibodies. **D**, **E** *Ppp6c*-KO cells were transduced with PPP6C or its phosphatase dead (PD) lentivirus and followed by the treatment with TZ for the indicated time. Cells were then stained with PI and analyzed under a microscope (**D**) or quantified by flow cytometer (**E**). Scale bar, 50 μm. **F**, **G** *Ppp6c*-KO L929 cells were transduced with PPP6C lentivirus and followed by the treatment with TZ for the indicated time. Cell lysates were resolved on SDS-PAGE (**F**) or non-reducing PAGE (**G**) for immunoblot with indicated antibodies. **H**, **I** *Ppp6c*-KO L929 cells were transduced with PPP6C or PPP6C-PD lentivirus and followed by the treatment with TZ for the indicated time. Cell lysates were resolved on non-reducing PAGE (**H**) or SDS-PAGE (**I**) for immunoblot with indicated antibodies. Data shown are representative of three independent experiments and presented as means ± SDs of triplicates (**C**, **E**). **p < 0.01, ***p < 0.001, with an unpaired Student’s *t*-test (**C**) or one-way ANOVA analysis (**E**).

PPP6C is the catalytic subunit of PP6, which exhibits multiple roles in regulating several cellular processes [29]. We were wondering whether its catalytic activity is required for its role in TNF-induced necroptosis. We first generated PPP6C-overexpressing L929 cells and found that overexpression of PPP6C sensitized L929 to TNF-induced necroptosis (Fig. 2A–C), which is consistent with its positive role in TNF-induced necroptosis. Then, we generated a PPP6C reintroducing (add-back) stable L929 cells (Re-PPP6C) by transducing *Ppp6c*-KO L929 cells with lentivirus HA-PPP6C. Of note, the protein level of add-back PPP6C was similar with the endogenous PPP6C protein (Fig. S2A). As expected, PPP6C re-expression restored the sensitivity of *Ppp6c*-KO L929 cells to TZ-induced necroptotic cell death (Fig. 2D, E), in conjunction with the expression of phosphorylated RIPK1 (S166), RIPK3 (S232) and MLKL (S345) (Fig. 2F), and the oligomeric MLKL (Fig. 2G). The catalysis of PPP6C requires several active residues including Asp84, associated with salt bridge formation and Arg85, related to phosphoryl group binding [41, 42]. We substituted Asp84 and Arg85 to Asn and Ala, respectively, to produce a D84N-R85A phosphatase-dead mutant (PPP6C-PD) (Fig. S2B). We found that only wild-type (WT) PPP6C, but not PPP6C-PD, restored the sensitivity of *Ppp6c*-KO L929 cells to TZ-induced necroptotic cell death indicated by PI staining (Fig. 2D, E). Consistently, the phosphorylation levels of RIPK1 (S166), RIPK3 (S232), and MLKL (S345), and MLKL oligomerization were increased in WT PPP6C but not PPP6C-PD re-expression L929 cells treated with TZ (Fig. 2H, I). Together, these results indicate that PPP6C regulates necroptosis depending on its phosphatase activity.

RIPK3 and MLKL are the key components in TNF-induced necroptotic signaling. We found that both the protein and mRNA levels of RIPK3 and MLKL were reduced in *Ppp6c*-KO L929 and *PPP6C* knockdown HT29 cells (Fig. S2A, C–E). As expected, re-expression of WT PPP6C but not PPP6C-PD in *Ppp6c*-KO L929 cells restored the expression of RIPK3 and MLKL (Fig. S2A, E). Our results suggest that *Ppp6c* depletion reduces the expression of RIPK3 and MLKL, which might partially contribute to the resistance to TNF-induced necroptosis.

### The resistance to TNF-induced necroptosis in *Ppp6c* deficient L929 cells is dependent on the TAK1-IKK signaling

PPP6C was previously revealed to oppose the activation of NF-κB at multiple steps [43, 44]. To elucidate the molecular mechanism by which PPP6C regulates necroptosis, we first investigated whether PPP6C regulates TNF-mediated NF-kB activity. By examining the nuclear translocation of P65, the phosphorylation levels of both IKKα/β and IκBα, and the responsive genes of NF-κB pathway, such as *A20*, *Tnf*, *IκB*, and *cIAP1*, we found that *Ppp6c*-KO L929 cells displayed a slightly faster and higher NF-κB activation compared with the control cells after TNF stimulation (Fig. S3A–D). In addition, we found that the expression of PPP6C suppressed NF-κB-driven luciferase activity induced by TNF, TAK1, and IKKα/β, but not by P65 (Fig. S3E). Thus, these data suggest that PPP6C represses TNF-induced NF-κB activity.

**Fig. 3:**
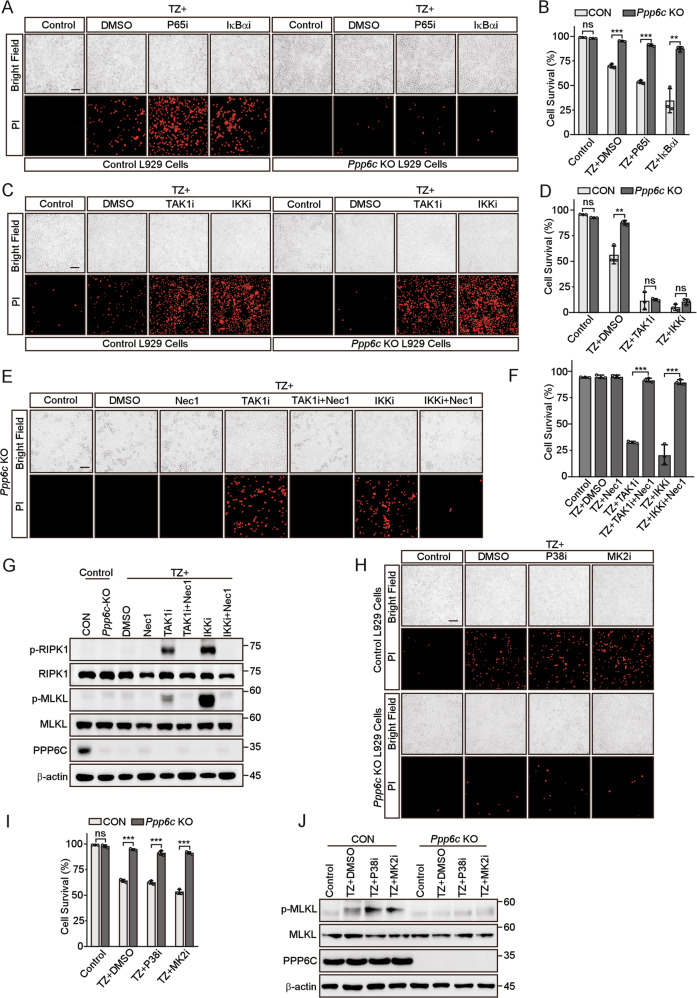
Inhibition of TAK1 or IKK activity restores TNF-induced necroptosis in *Ppp6c* deficient L929 cells. **A**, **B** Control and *Ppp6c*-KO L929 cells were pretreated with P65 inhibitor (Maslinic acid, 20 μM) or IκBα inhibitor (BAY117085, 10 μM) for 30 min and then treated with TZ for 4 h. Cells were stained with PI, and analyzed under a microscope (**A**) or quantified by flow cytometer (**B**). Scale bar, 50 μm. (**C**, **D**) Control and *Ppp6c*-KO L929 cells were pretreated with TAK1 inhibitor (5Z-7-Oxozeaenol, 1 μM) or IKKα/β inhibitor (IKK16, 1 μM) for 30 min and then treated with TZ for 4 h. Cells were stained with PI, and analyzed under a microscope (**C**) or quantified by flow cytometer (**D**). Scale bar, 50 μm. **E**–**G** *Ppp6c*-KO L929 cells were pretreated with TAK1 inhibitor (5Z-7-Oxozeaenol, 1 μM) or IKKα/β inhibitor (IKK16, 1 μM) in the presence or absence of Necrostatin-1 (10 μM) for 30 min, and then treated with TZ for 4 h. Cells were stained with PI and analyzed under a microscope (**E**) or quantified by flow cytometer (**F**). Cell lysates were probed with indicated antibodies (**G**). Scale bar, 50 μm. **H**–**J** Control and *Ppp6c*-KO L929 cells were pretreated with P38 inhibitor (Adezmapimod, 10 μM) or MK2 inhibitor (MK2-IN-1, 10 μM) for 30 min, and then treated with TZ for 4 h. And cells were stained with PI and analyzed under a microscope (**H**) or quantified by flow cytometer (**I**), and cell lysates were probed with indicated antibodies (**J**). Scale bar, 50 μm. Data shown are representative of three independent experiments and presented as means ± SDs of triplicates (**B**, **D**, **F**, **I**). **p < 0.01, ***p < 0.001, with an unpaired Student’s *t*-test (**B**, **D**, **I**) or one-way ANOVA analysis (**F**).

To evaluate whether NF-κB activity is required for PPP6C-regulated necroptosis, we treated L929 cells with several chemical inhibitors targeting the components in NF-κB pathway together with TZ. We found that blocking NF-κB pathway by two different inhibitors, maslinic acid (targeting P65) [45] and BAY 11-7082 (targeting IκBα) [46], significantly increased cell death in control cells but had little effect on *Ppp6c*-KO L929 cells (Fig. 3A, B, Fig. S3F). Intriguingly, TAK1 inhibitor 5Z-7-Oxozeaenol [47] or IKKα/β inhibitor IKK16 [48] completely abolished the resistance of *Ppp6c*-KO cells to TZ-induced necroptosis (Fig. 3C, D, Fig. S3G). Co-treatment with the RIPK1 inhibitor Necrostatin-1 (Nec1) fully reversed this sensitization to necroptosis induced by TAK1 and IKK inhibitors in *Ppp6c*-KO L929 cells (Fig. 3E–G). TAK1 has been reported to phosphorylate RIPK1 at S320/321 directly, or indirectly via its downstream pathway such as P38-MK2, which inhibits RIPK1 kinase activity and prevents necroptosis [24–26]. To test whether P38-MK2 signaling cascade is involved in the resistance to TZ-induced necroptosis in *Ppp6c*-KO L929 cells, we treated the cells with TZ in the presence of adezmapimod or MK2-IN-1, which inhibits P38 and MK2 respectively to prevent TNF-induced P38 and MK2 activation. We found that pharmacologic inhibition of P38 or MK2 did not interfere with the resistance to TZ-induced necroptosis in *Ppp6c*-KO L929 cells (Fig. 3H–J, Fig. S3H). Together, our results suggest that the resistance to TZ-induced necroptosis in *Ppp6c*-KO L929 cells is dependent on the TAK1-IKK signaling activity, but not P38-MK2, or IκBα-P65 signaling cascade.

### PPP6C regulates necroptosis by dephosphorylating TAK1

**Fig. 4:**
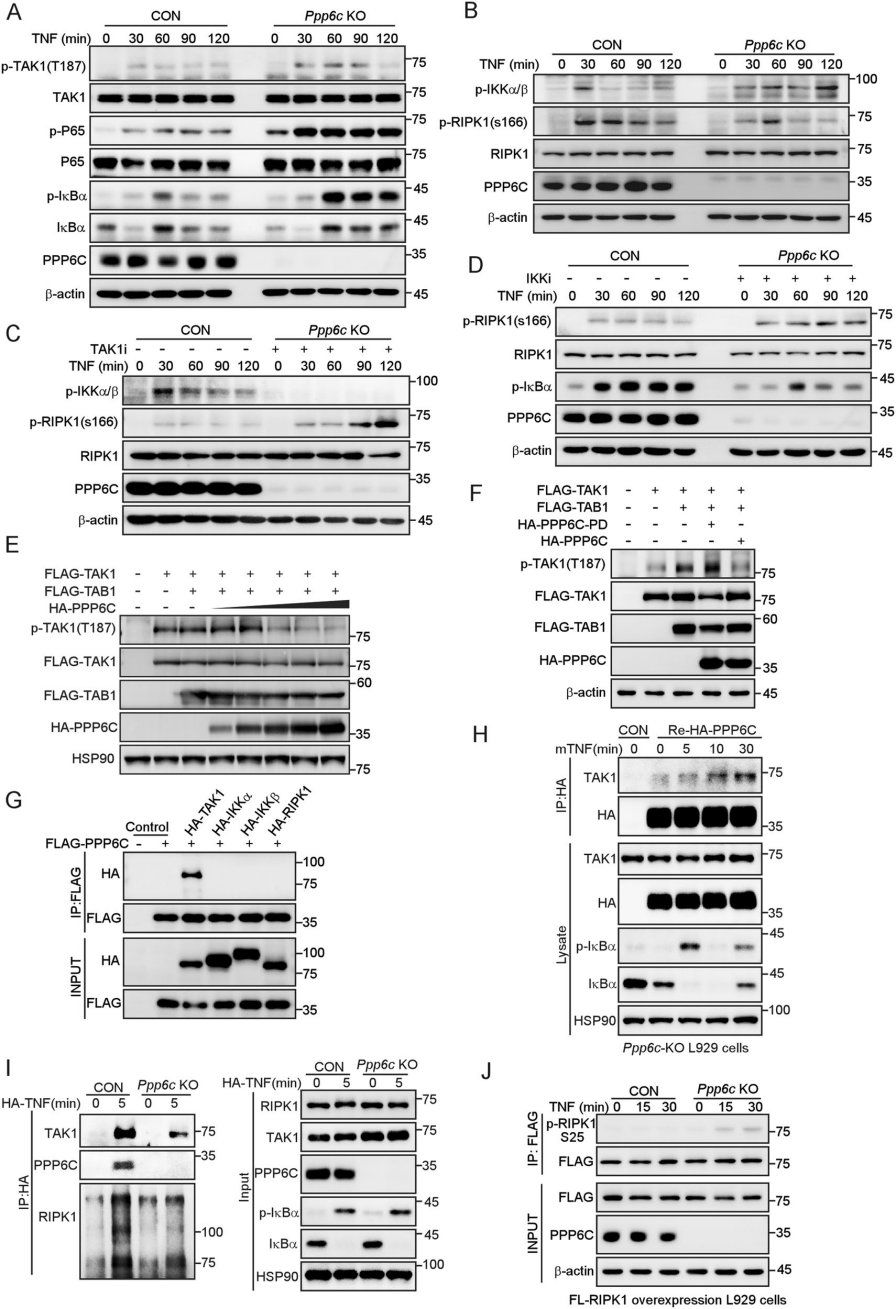
PPP6C interacts with and dephosphorylates TAK1. **A**, **B** Control and *Ppp6c*-KO L929 cells were treated with TNF (10 ng/ml) for the indicated time, and then lysed for immunoblot with indicated antibodies. **C**, **D** Control and *Ppp6c*-KO L929 cells were pretreated with TAK1 inhibitor (5Z-7-Oxozeaenol, 1 μM) (**C**) or IKKα/β inhibitor (IKK16, 1 μM) (**D**) for 30 min and then treated with TNF (10 ng/ml) for the indicated time. Cell lysates were probed with indicated antibodies. **E** HEK293T cells were transfected with TAK1 and TAB1 plasmids together with different dose of PPP6C plasmid. Cell lysates were probed with indicated antibodies 24 h after transfection. **F** HEK293T cells were transfected with TAK1 and TAB1 plasmids together with PPP6C or PPP6C-PD mutant plasmids. Cell lysates were probed with indicated antibodies 24 h after transfection. **G** HEK293T cells were transfected with the indicated plasmids and then lysed for co-immunoprecipitation assay as indicated. **H** *Ppp6c*-KO L929 cells were transduced with HA-PPP6C lentivirus and then treated with TNF (10 ng/ml) for the indicated time, and lysed for co-immunoprecipitation assay as indicated. **I** Control and *Ppp6c*-KO L929 cells were stimulated with 1 μg/ml HA-mTNF for 5 min. TNFR1 complex I was immunoprecipitated by anti-HA antibody and analyzed by immunoblotting. **J** Control and *Ppp6c*-KO L929 cells were transduced with FLAG-RIPK1 lentivirus and then treated with TNF (10 ng/ml) for the indicated time. FLAG-RIPK1 proteins were immunoprecipitated with anti-FLAG antibody and probed with anti-p-RIPK1 (S25) antibody.

It has been reported that PPP6C negatively regulates IL-1 signaling via dephosphorylating TAK1 at T187 [31], a key phosphorylation site that determines whether TAK1 is activated [49]. To dissect the molecular mechanism that causes the resistance to TZ-induced necroptosis in *Ppp6c*-KO L929 cells, we examined the phosphorylation status of TAK1. The result revealed that the phosphorylation level of TAK1 at T187 was enhanced and sustained longer in *Ppp6c*-KO L929 cells in response to TNF stimulation (Fig. 4A). In addition, we found a prolonged IKKα/β activation, and a lower activity of RIPK1 indicated by the reduced S166 phosphorylation in *Ppp6c*-KO L929 cells following TNF stimulation (Fig. 4B). Thus, these results imply that *Ppp6c* depletion impairs the RIPK1 kinase activity in TNF-treated L929 cells. To further explore whether the inhibition of RIPK1 kinase activity is dependent on TAK1-IKKα/β axis, we made use of pharmacologic inhibitors. We found that the inhibition of TAK1 or IKKα/β significantly increased the phosphorylation of RIPK1 at S166 in TNF-treated *Ppp6c*-KO L929 cells (Fig. 4C, D), while the inhibition of IκBα or P65 had no such effect (Fig. S4A, B). These results indicate that RIPK1 kinase activity is regulated by TAK1-IKKα/β cascade in *Ppp6c*-KO L929 cells.

TAK1 was reported as a substrate of PPP6C [31]. Consistent with previous finding, PPP6C overexpression reduced the phosphorylation of TAK1 at T187 in a dose-dependent manner (Fig. 4E), and the phosphatase dead mutant of PPP6C had no such activity (Fig. 4F). In addition, co-immunoprecipitation assay showed that PPP6C was associated with TAK1 but not IKKα/β or RIPK1 (Fig. 4G), implying that PPP6C may regulate RIPK1 kinase activity through TAK1, rather than by dephosphorylating IKKα/β or RIPK1. To investigate whether the interaction between PPP6C and TAK1 was regulated by TNF stimulation, we re-expressed HA-PPP6C in *Ppp6c*-KO L929 cells and performed a co-immunoprecipitation assay. The result showed that the endogenous TAK1 was associated with HA-PPP6C and the interaction was enhanced by TNF treatment in L929 cells (Fig. 4H). Importantly, we found that endogenous PPP6C was recruited to TNFR1 complex under TNF stimulation in L929 cells, and TAK1 was marked reduced in TNFR1 complex I when *Ppp6c* was depleted in L929 cells (Fig. 4I). Previous research found that RIPK1 is a direct substrate of IKKα/β [22], and IKKα/β phosphorylate RIPK1 at S25 to prevent RIPK1 kinase activation [27]. In accordance with a higher IKKα/β activity, enhanced phosphorylation of RIPK1 at S25 was detected in *Ppp6c*-KO L929 cells upon TNF treatment (Fig. 4J). To examine whether PPP6C regulates the activity of IKKα/β or RIPK1, we used *TAK1*-KO HEK293T cells to rule out the interference of TAK1 on the activity of IKKα/β or RIPK1. We transfected *TAK1*-KO HEK293T cells with IKKα/β, RIPK1, and PPP6C plasmids and found that PPP6C has little effect on the phosphorylation levels of IKKα/β or RIPK1 when TAK1 is deficient (Fig. S4C). As a core regulator of TNF-induced necroptosis, RIPK1 can be phosphorylated at multiple sites, whether PPP6C could dephosphorylate RIPK1 at other IKKα/β-independent sites needs further investigation. Taken together, these data suggest that *Ppp6c* deficiency inhibits RIPK1 kinase activity, at least in part, through acting on TAK1-IKKα/β signaling axis to increase phosphorylation of RIPK1 at S25, which ultimately prevents TNF-induced necroptosis.

### TAB2 is essential for the resistance to TNF-induced necroptotic cell death in *Ppp6c* deficient cells

**Fig. 5:**
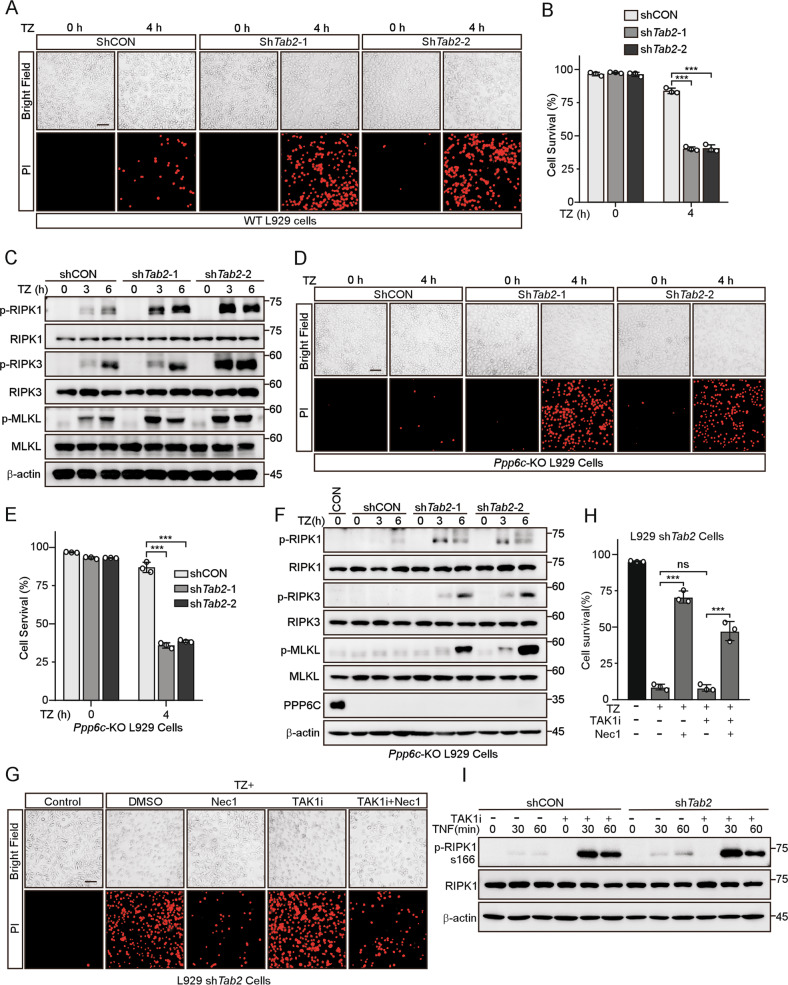
TAB2 is required for the resistance to TNF-induced necroptotic cell death in PPP6C deficient cells. **A**, **B** Control and *Tab2*-knockdown L929 cells were treated with TZ for the indicated time, and analyzed under a microscope (**A**) or quantified by flow cytometer (**B**) after stained with PI. Scale bar, 50 μm. **C** Control and *Tab2*-knockdown L929 cells were treated with TZ for the indicated time and then lysed for immunoblot with indicated antibodies. **D**, **E** Control and *Tab2*-knockdown *Ppp6c*-KO L929 cells were treated with TZ for the indicated time, and analyzed under a microscope (**D**) or quantified by flow cytometer (**E**) after stained with PI. Scale bar, 50 μm. **F** Control and *Tab2*-knockdown *Ppp6c*-KO L929 cells were treated with TZ for the indicated time and then lysed for immunoblot with indicated antibodies. **G**, **H** Control and *Tab2*-knockdown L929 cells were pretreated with TAK1 inhibitor (5Z-7-Oxozeaenol, 1 μM) in the presence or absence of Necrostatin-1 (10 μM) for 30 min, and then treated with TZ for 4 h. Cells were stained with PI and analyzed under a microscope (**G**) or quantified by flow cytometer (**H**). Scale bar, 50 μm. **I** Control and *Tab2*-knockdown L929 cells were pretreated with TAK1 inhibitor (5Z-7-Oxozeaenol, 1 μM) for 30 min, and then treated with TNF (10 ng/ml) for the indicated time. Cell lysates were probed with indicated antibodies. Data shown are representative of three independent experiments and presented as means ± SDs of triplicates (**B**, **E**, **H**). **p < 0.01, ***p < 0.001, with one-way ANOVA analysis (**B**, **E**, **H**).

A previous study has shown that TAK1-binding protein 2 (TAB2), is necessary for TAK1 inactivation by recruiting PPP6C to the TAK1 complex [50] and deficiency in TAB2 leads to sustained TAK1 activation but unexpectedly sensitized cells to necroptosis [28]. To investigate whether TAB2 involves in the resistance to necroptosis in *Ppp6c*-KO L929 cells, we used shRNA to deplete *Tab2* in WT and *Ppp6c*-KO L929 cells. The knockdown efficiency was determined by quantitative RT-PCR (Fig. S5A). Consistent with the previous report, we found that *Tab2*-depleted L929 cells had elevated TAK1 phosphorylation (Fig. S5B) and were more sensitive to TNF-induced necroptosis (Fig. 5A–C, Fig. S5C). However, to our surprise, knockdown of *Tab2* also remarkably increased TNF-induced necroptosis in *Ppp6c*-KO L929 cells (Fig. 5D–F). These results suggest that *Tab2* depletion abolished the resistance to necroptosis in *Ppp6c*-KO L929 cells. Since both *Ppp6c* and *Tab2* depletion resulted in the increased TAK1 activity, but led to opposite effect on TZ-induced necroptosis. To further investigate this, we stimulated cells with TNF, and found that TAK1 and IKKα/β were sustainably activated in *Tab2*-depletion cells just like that in *Ppp6c*-KO L929 cells. If the increased necroptotic cell death in *Tab2*-depleted cells was caused by hyperactivation of TAK1, one would expect that inhibition of TAK1 would reduce TNF-induced necroptotic cell death in *Tab2*-depleted cells. However, inhibition of TAK1 by 5Z-7-Oxozeaenol did not prevent TNF-induced necroptotic cell death in *Tab2*-depleted cells (Fig. 5G, H). In addition, the increased RIPK1 kinase activity indicated by the phosphorylation level at S166 of RIPK1 in *Tab2-*depleted cells was not affected by TAK1 inhibition during TNF stimulation (Fig. 5I). Together, these results suggest that TAB2 may play a more complicated regulatory role in the necroptosis signaling and depletion of *Tab2* sensitizes *Ppp6c*-KO cells to TNF-induced necroptosis.

### *Ppp6c* loss alleviates necroptosis-related tissue injury and inflammation

**Fig. 6:**
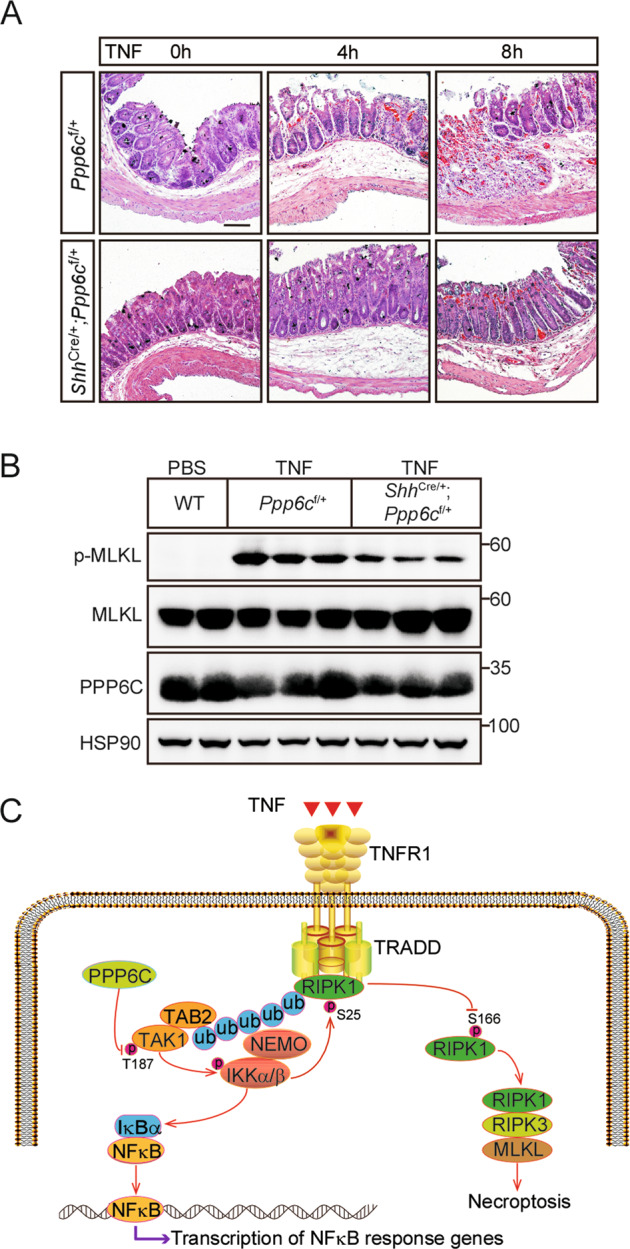
PPP6C loss alleviates TNF-induced necroptosis in SIRS model. **A** Representative H&E staining of cecum from *Ppp6c*^f/+^ and *Shh*^*Cre/+*^;*Ppp6c*^f/+^ mice (*n* = 3 each group at a time point) injected with TNF for the indicated time. Scale bar, 100 μm. **B** Cecal proteins from *Ppp6c*^f/+^ and *Shh*^*Cre/+*^;*Ppp6c*^f/+^ mice injected with TNF for 8 h were analyzed by immunoblot with indicated antibodies. **C** A schematic model shows that PPP6C positively regulates TNF-induced necroptosis via TAK1-IKKα/β axis.

Necroptosis is an inflammatory form of programmed cell death and dysregulated necroptosis leads to intestinal inflammation [51]. It has been demonstrated that necroptosis is essential for TNF-induced SIRS, as both *Ripk3*^*-/-*^ and Mlkl^-/-^ mice are protected from TNF-induced SIRS [52, 53]. We utilized a TNF-SIRS mouse model to determine whether PPP6C regulates the onset of systemic inflammatory response syndrome. It is important to note that the cecum was described as particularly sensitive to TNF-induced injury in the TNF-SIRS model [54], so we used *Shh*^*Cre*^ driver to delete *Ppp6c* in gastrointestinal tract. Lineage tracing study from *Shh*^*Cre/+*^*;Rosa26*^*tdTomato*^ mice confirmed the Cre activity in mouse gastrointestinal tract (Fig. S6A). We crossed the *Shh*^*Cre/+*^ mice with *Ppp6c*^*flox/flox*^ mice to obtain *Ppp6c* intestine knockout mice. However, it is likely that *Shh*^*Cre/+*^*-*mediated *Ppp6c* knockout mice are embryonic lethal, we did not obtain *Shh*^*Cre/+*^*;Ppp6c*^*flox/flox*^ offspring. Therefore, *Shh*^*Cre/+*^*;Ppp6*^*flox/+*^ mice were used for further investigation. Their littermates, *Ppp6c*^*flox/+*^ mice, were used as the control. We found that *Shh*^*Cre/+*^*;Ppp6*^*flox/+*^ mice showed partial protection from TNF-induced cecum damage compared with the littermate control mice after intravenous injection of mouse TNF (Fig. 6A). In addition, analysis of the cecum lysate showed that TNF-induced phosphorylation of MLKL in control mice was higher than that in *Shh*^*Cre/+*^*;Ppp6*^*flox/+*^ mice (Fig. 6B). These data further support that loss of PPP6C prevent TNF-induced cecum injury through inhibition of necroptosis. In addition, we employed a DSS-induced colitis mouse model to further investigate the role of PPP6C in regulating the inflammatory diseases. We found that upon DSS treatment, *Shh*^*Cre/+*^*;Ppp6c*^*flox/+*^ mice showed less weight loss compared to the control (Fig. S6D), and markedly longer colons than that in DSS-treated control mice (Fig. S6B, C, E, F). Consistently, H&E staining also revealed less inflammation and mucosal epithelium damage in *Shh*^*Cre/+*^*;Ppp6c*^*flox/+*^ mice (Fig. S6G). Consistent with TNF-SIRS model, MLKL was less phosphorylated in *Shh*^*Cre/+*^*;Ppp6c*^*flox/+*^ mice (Fig. S6H), indicating that the necroptotic pathway was compromised in *Shh*^*Cre/+*^*;Ppp6c*^*flox/+*^ mice. Moreover, reduced apoptotic cell death of intestinal cells was found in DSS-treated *Shh*^*Cre/+*^*;Ppp6c*^*flox/+*^ mice by TUNEL staining (Fig. S6I). It is worth noting that whether necroptosis or the kinase activity of RIPK1 is necessary for DSS-induced colitis is still debatable [53, 55], so we speculate that PPP6C might play a more complex role in regulating tissue injury and inflammation beyond its ability to promote necroptosis. Collectively, these results indicate that *Ppp6c* depletion alleviates necroptosis-related tissue injury and inflammation.

## Discussion

In this study, we performed a positive-selection screen to identify necroptosis regulators in L929 cells by using CRISPR/Cas9 gene knockout library against 20,611 genes of the mouse genome and identified 79 genes by positive sgRNA enrichment. As expected, we found a strong enrichment of sgRNA targeting TNF receptor *Tnfrsf1a* and known necrosome components *Ripk1*, *Ripk3*, *Mlkl* and some moderate enrichments of sgRNAs targeting genes related to RIPK1 posttranslational modification, such as *Cyld* and *Spata2*. In addition, we identified *Ppp6c* as a novel mediator of necroptosis. Intriguingly, we noticed some similar screens for necroptosis resistance were previously performed in several publications [35, 56–62], but there is a remarkable lack of overlap between the regulators identified in those genetic screens except a few key factors such as *Ripk1*, *Ripk3*, and *Mlkl*. Similarly, little overlap of screen hits exists between our study and other reported screens. We suspect that those differences in screening strategies among different groups might partly contribute to the discrepancy of screen hits. So different screening strategies may complement each other and help us get a more complete understanding of necroptosis. More importantly, the nonoverlapping hits among many of these screens also suggest that some of the regulators influence necroptosis in a context-dependent manner except the core RIPK1-RIPK3-MLKL axis.

Necroptosis is a process tightly regulated by a variety of precise phosphorylation, and its key regulators RIPK1, RIPK3, and MLKL are all phosphorylated at multiple positions. Many kinases such as TAK1, MK2, IKKα/β, CK1, and TAM [24, 25, 27, 63, 64] were previously reported to play a crucial role in the necroptosis signaling pathway. However, how the necroptosis is regulated by the phosphatase which acts on the opposition to the kinase is still rarely reported. In this study, we revealed that *Ppp6c* is a novel positive regulator of necroptosis by genome-wide CRISPR/Cas9 library screening. We showed that the deficiency of *Ppp6c* protects cells from TNF-induced necroptotic cell death. Accordingly, deletion of *Ppp6c* increases TNF-induced IKKα/β activation which inhibits RIPK1 kinase activity. TNF-induced necroptotic cell death could be restored by TAK1 or IKKα/β inhibitor instead of NF-κB inhibitor in *Ppp6c*-KO L929 cells. Although we showed that PPP6C interacts with and dephosphorylates TAK1, we could not rule out the possibility that PPP6C acts directly on RIPK1 which is phosphorylated at multiple sites. Thus, our study reveals the molecular mechanism how PPP6C controls the activity of RIPK1 kinase and regulates TNF-induced necroptosis, at least in part, via the TAK1-IKKα/β cascade (Fig. 6C), suggesting that the phosphatase also plays important roles in the process of necroptosis.

Global deletion of *Ppp6c* leads to embryonic lethality in mice [40], suggesting that *Ppp6c* plays essential functions during the normal development. PPP6C has been implicated in DNA damage repair, oocyte, and lymphocyte development, virus infection, innate immune responses, carcinogenesis, etc [29, 65, 66]. Here, we showed that the colon epithelium-specific knockdown of *Ppp6c* alleviates necroptosis-related tissue injury and inflammation. Interestingly, we found *Ppp6c* deletion reduced cell proliferation in MEFs while deletion of *Ppp6c* in L929 cells had no such effect. Similarly, one study reported that shRNA-mediated *PPP6C* knockdown in HepG2 cells has little influence on proliferation rate [67], but another research showed that in vivo infection of lentivirus shRNA targeting *Ppp6c* in epidermis leads to an enhanced proliferation of keratinocyte [68]. As a multifunctional phosphatase, PPP6C has diverse important functions and controls various physiological processes. A functional PP6 holoenzymes usually consists of its catalytic subunit (PPP6C) and other regulatory subunits. Its diverse functions may be regulated by these regulatory subunits.

We found that the depletion of *Ppp6c* in MEF cells only delays necroptotic cell death and *Ppp6c*-KO MEF cells eventually died after longtime exposure to TNF. We postulate that there might be other phosphatases that play a compensatory role in the absence of *Ppp6c* in MEF cells. As an evolutionarily conserved and ubiquitously expressed protein phosphatase, PP6 is highly homologous with protein phosphatase 2A (PP2A) and protein phosphatase 4 (PP4) [29], however, PP6 has received less attention than its close relative PP2A. Since PP6 is closely related to PP2A and PP4, whether PP2A or PP4 has a similar regulatory function on necroptosis is worthy of further investigation. Noteworthy, PPM1A and PPM1B were identified as IKKβ phosphatases to synergistically terminate TNF-induced IKKβ and NF-κB activation [69]. Only PPM1B was showed to negatively regulate necroptosis through dephosphorylating RIPK3 while PPM1A did not regulate necroptosis [70]. These studies indicate that two close phosphatases with similar structures may still not have an overlap function. Further studies are needed to determine the mechanism of phosphatases in the regulation of cell death.

TAK1 is considered as a pro-survival regulator via promoting the activation of NF-κB pathway [23]. Accumulating evidence suggests that TAK1 also regulates apoptosis and necroptosis pathway independent on its role in NF-κB activation. PPP6C was reported to be recruited to the TAK1 complex in a TAB2-dependent manner [50]. Similar to PPP6C deletion, deficiency of TAB2 leads to sustained TAK1 activation. However, TAB2 deficiency sensitized cells to necroptosis [28], which is opposite to PPP6C inactivation. Our data showed that TAB2 is essential for *Ppp6c*-KO cells to survive under TZ treatment, since *Tab2* knockdown abolished the *Ppp6c* deletion-mediated resistance to TNF-induced necroptosis. Our data suggest that *Tab2* knockdown promotes the activation of both TAK1 and RIPK1, and leads to RIPK1 kinase-dependent necroptosis. Considering the fact that *Ppp6c* removal leads to prolonged TAK1 activation but still protects cells from necroptosis, we speculate that TAK1 hyperactivation in *Tab2* knockdown cells does not necessarily lead to hypersensitivity to TNF-induced necroptosis. It is possible that the deficiency of *Tab2* promotes RIPK1 kinase activity and leads to necroptosis in a TAK1-independent manner. Intriguingly, in contrast to the previous finding and ours, a recent work showed that ablation of *Tab2* blocks, instead of promotes, TAK1 activation in cardiomyocytes [71]. It is likely that the discrepancy is caused by different cell types and warrants further investigation.

In summary, the previous studies have revealed that a variety of kinases play essential roles in regulating necroptosis, while the functions of phosphatases were rarely studied in the pathway. Herein, we identified PPP6C as a positive regulator of necroptosis, and revealed a previously unknown function of PPP6C in regulating programed cell death.

## Supplementary information

aj-checklist

Uncropped blot

supplementary figure legend and tables CDDIS-22-0857R2

Figure S1

Figure S2

Figure S3

Figure S4

Figure S5

Figure S6

## Data Availability

Materials are available from the corresponding author upon reasonable request.
