# Supplementary material for: Deficiency of PPP6C protects TNF-induced necroptosis through activation of TAK1
Source: Cell Death Dis. 2022 Jul 16;13(7):618. doi: 10.1038/s41419-022-05076-1 (PMC9288536; doi:10.1038/s41419-022-05076-1)

Uncropped blot images of the indicated Figures (page 1 of 5)

Fig. 1E

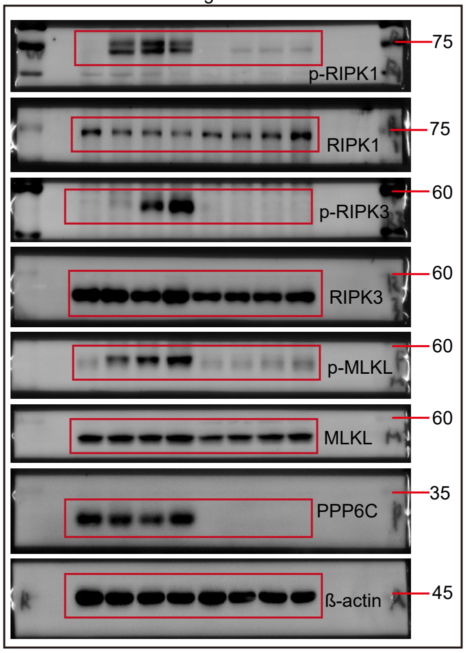

Fig. 1I

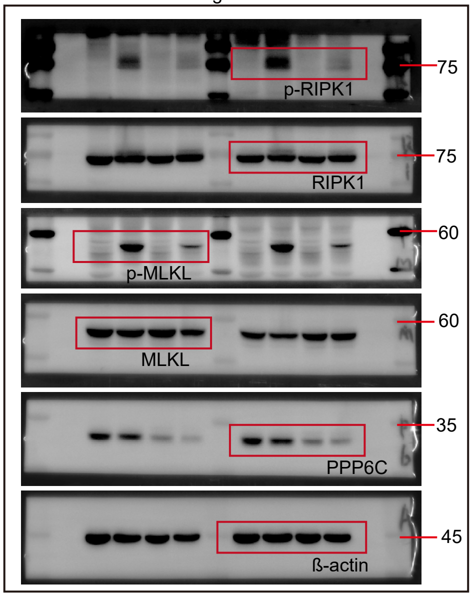

Fig. 2F

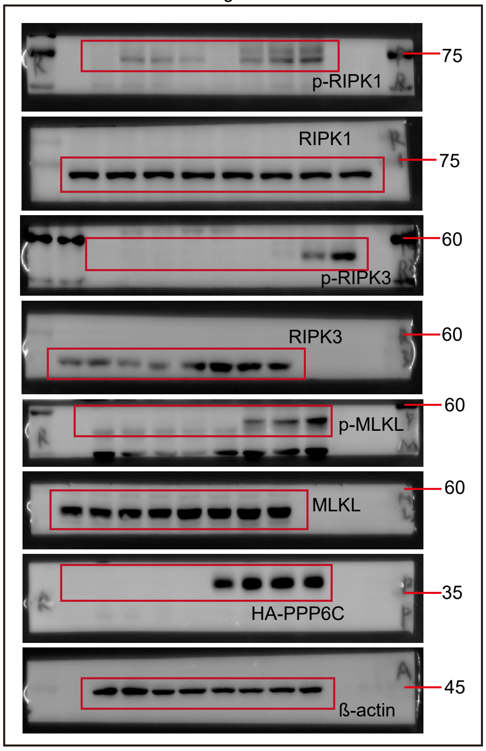

Fig. 1F

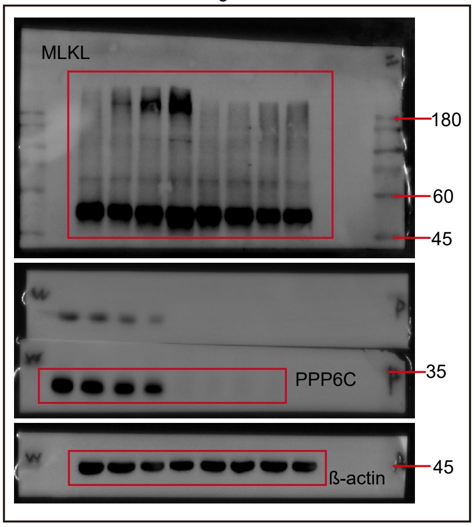

Fig. S1F

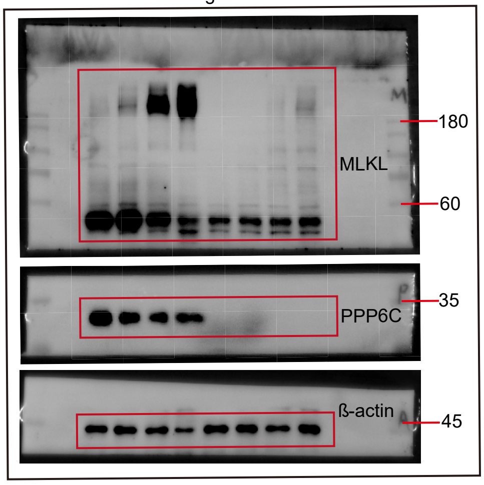

Fig. 2B

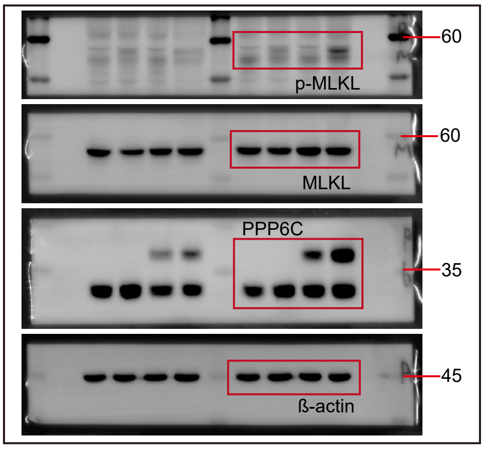

Fig. 2G

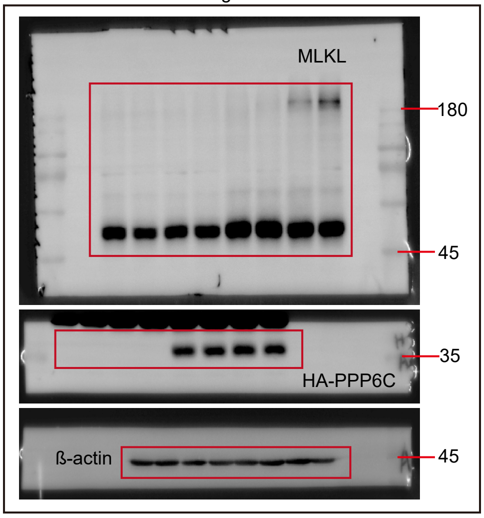

Fig. 1G

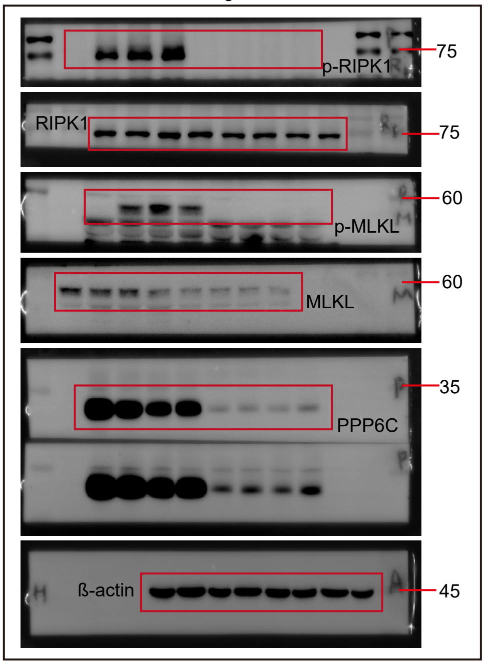

Fig. 2H

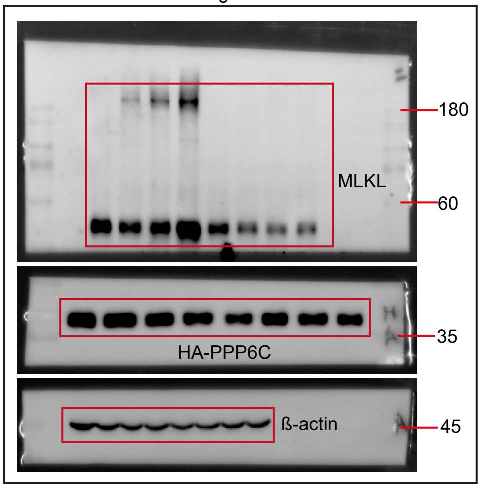

Fig. 2I

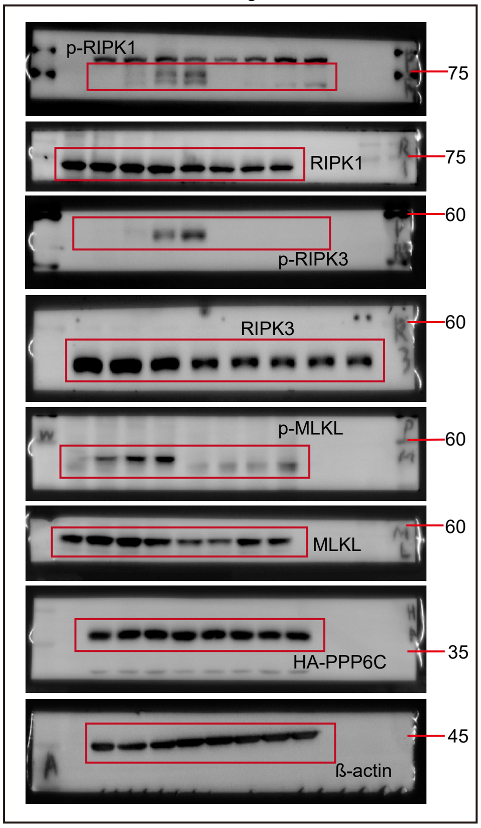

Uncropped blot images of the indicated Figures (page 2 of 5)

Fig. S2A

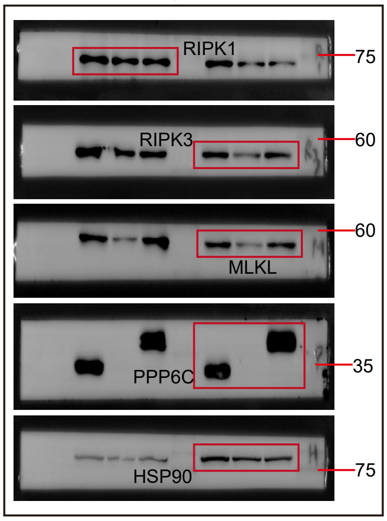

Fig. S2C

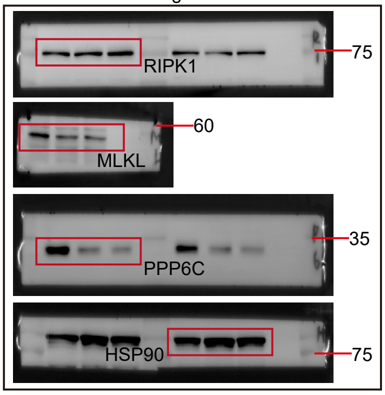

Fig. 3G

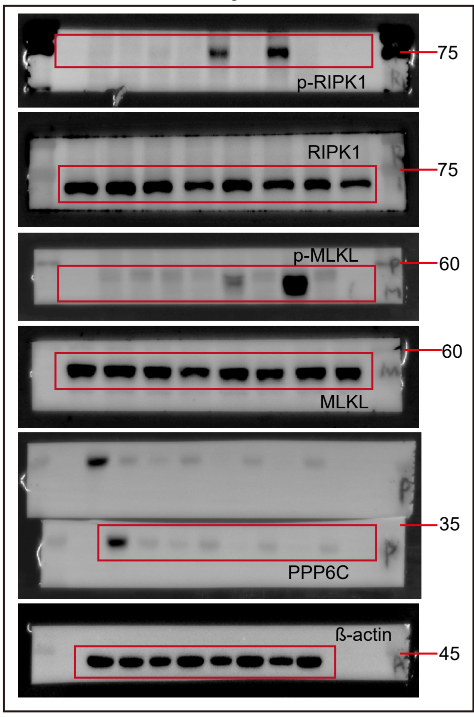

Fig. 3J

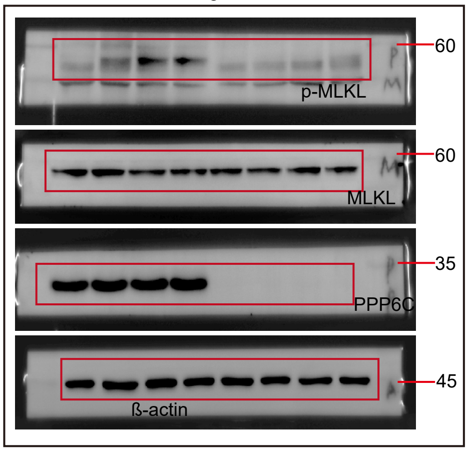

Fig. S3C

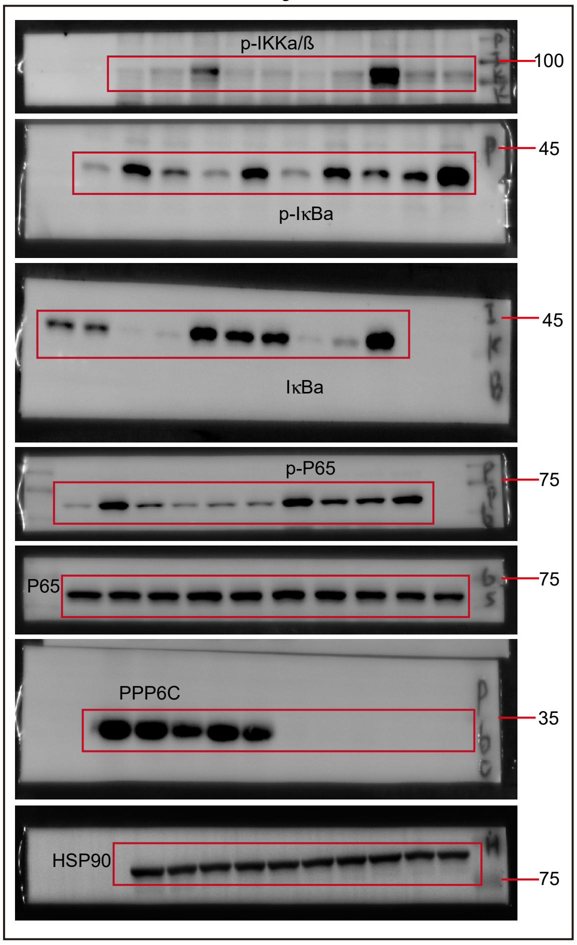

Fig. S3G

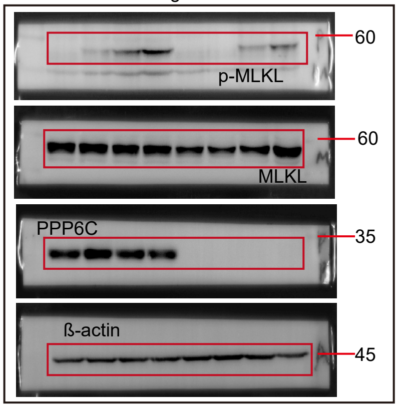

Fig. S3F

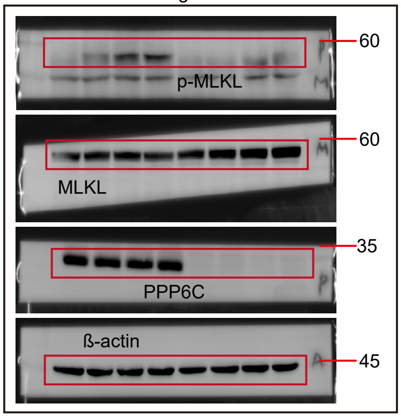

Fig. S3E

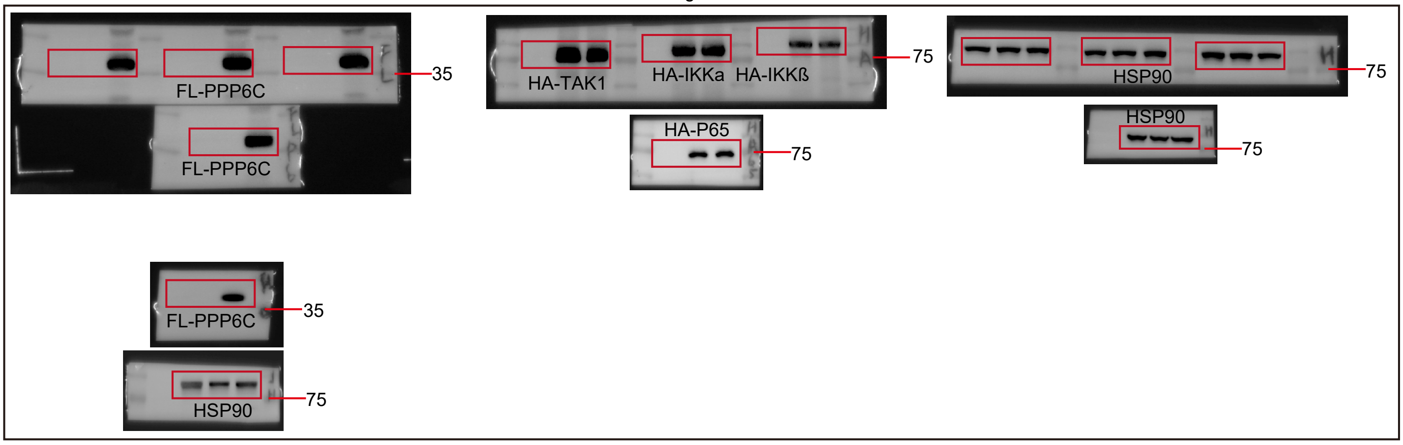

Uncropped blot images of the indicated Figures (page 3 of 5)

Fig. S3H

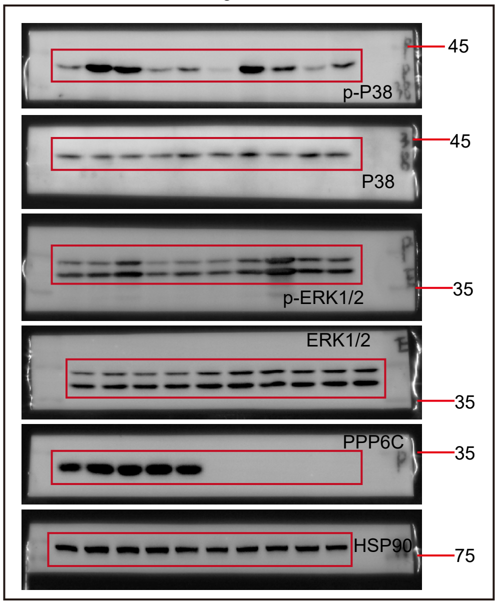

Fig. 4A

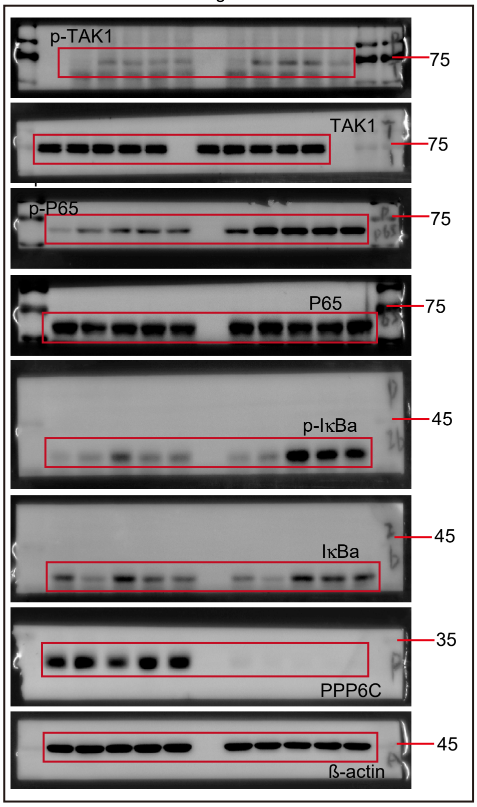

Fig. 4B

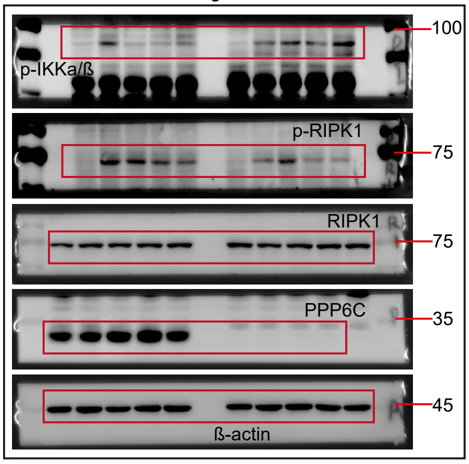

Fig. 4C

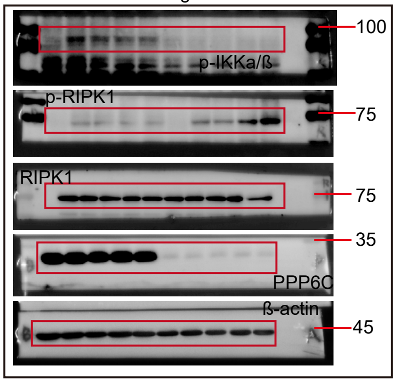

Fig. 4D

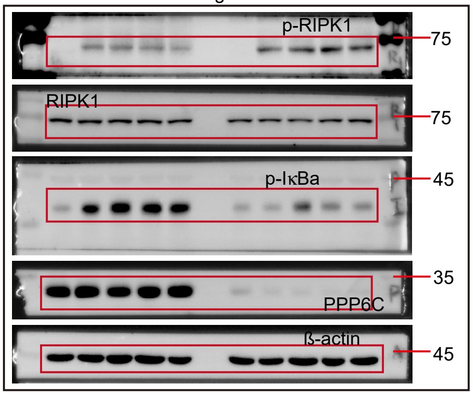

Fig. 4E

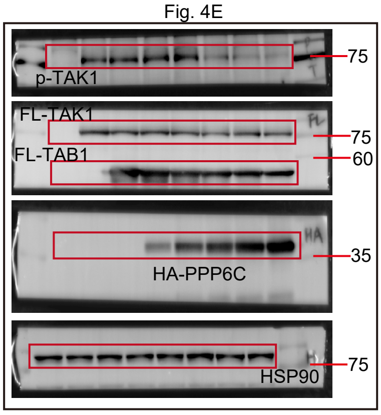

Fig. 4F

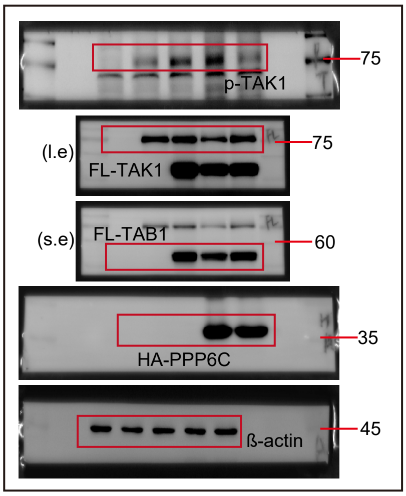

Fig. 4G

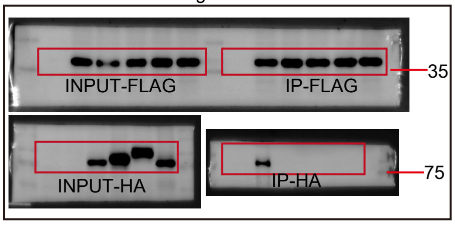

Fig. 4H

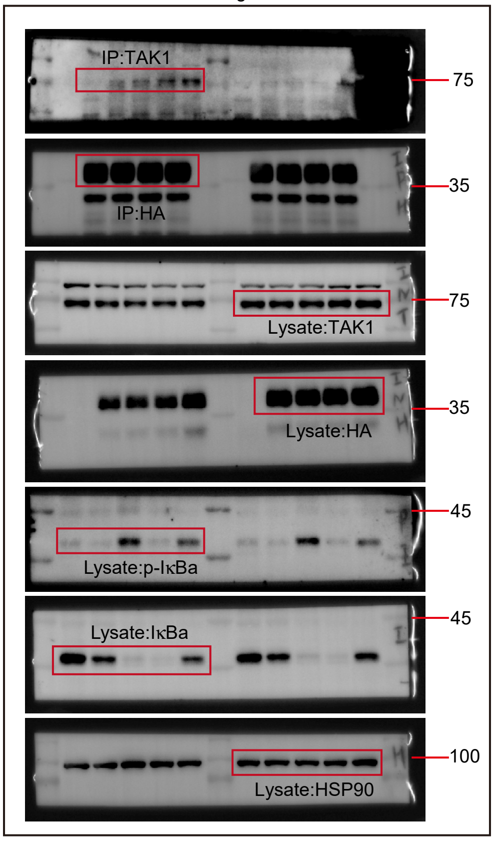

Fig. 4I

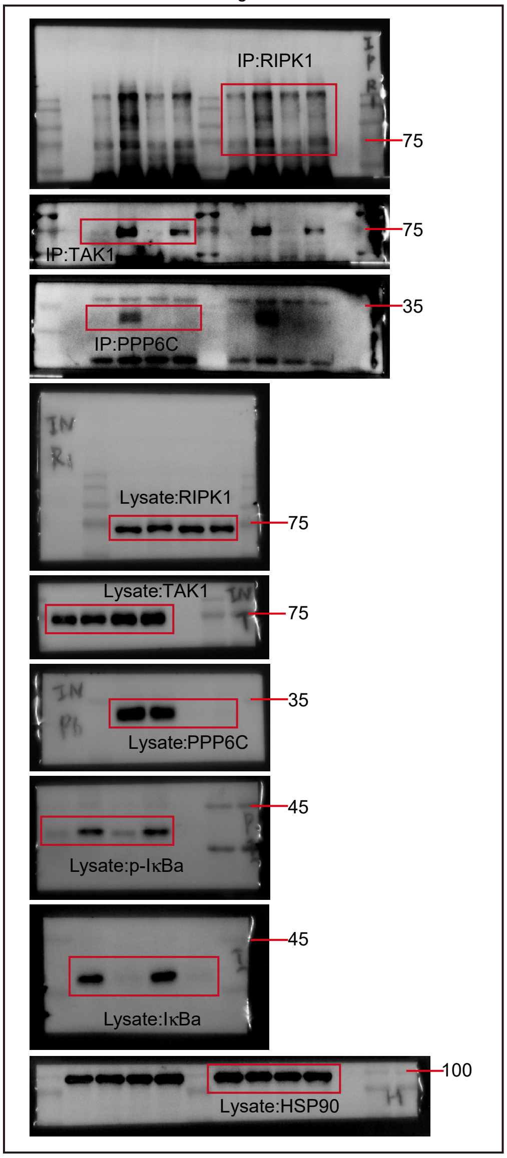

Uncropped blot images of the indicated Figures (page 4 of 5)

Fig. 4J

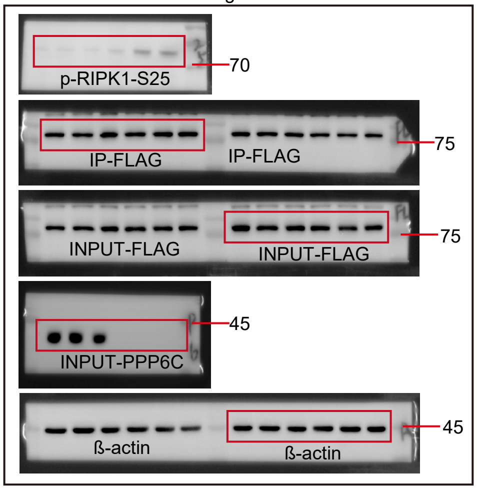

Fig. S4A

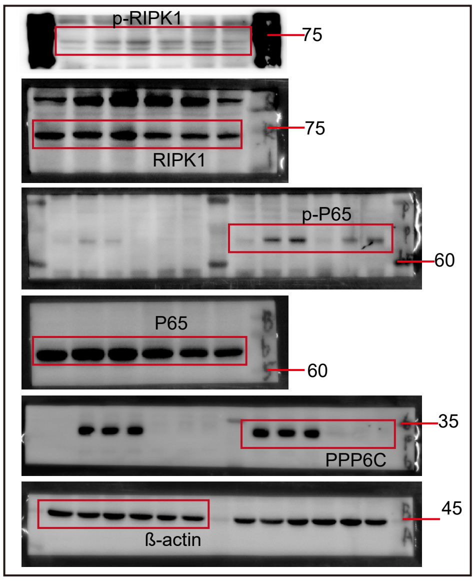

Fig. S4B

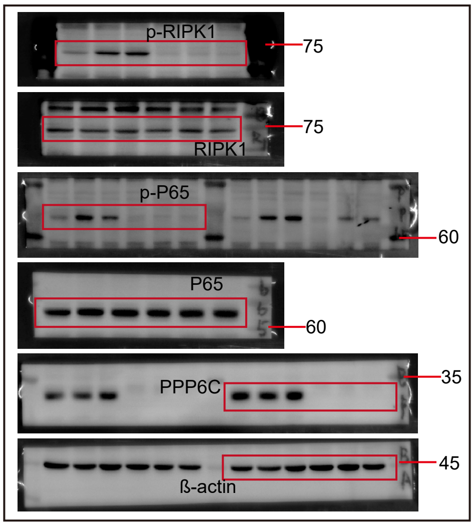

Fig. S4C

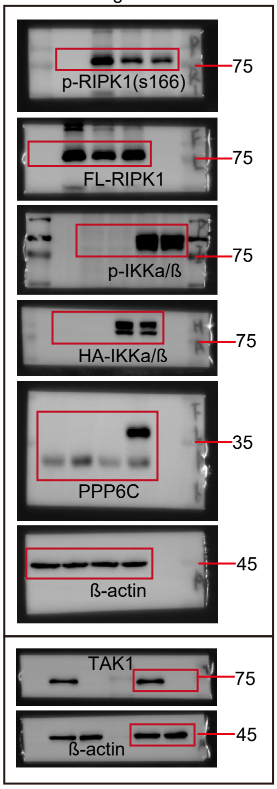

Fig. 5C

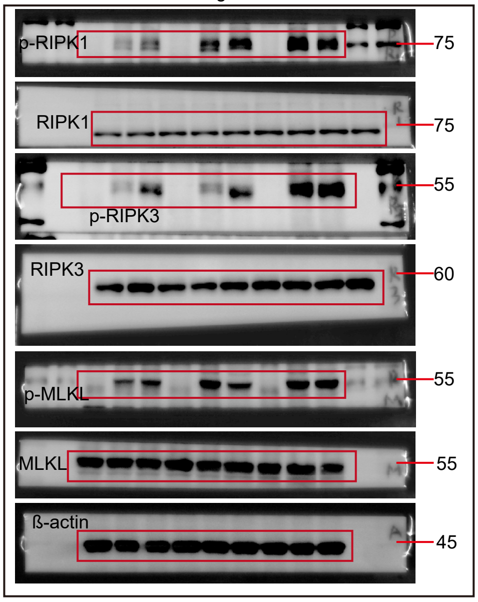

Fig. 5I

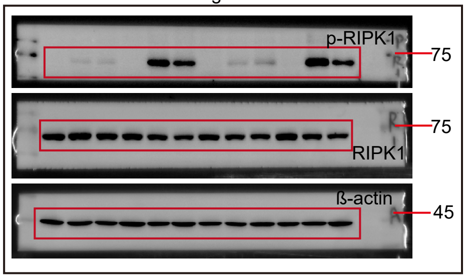

Fig. 5F

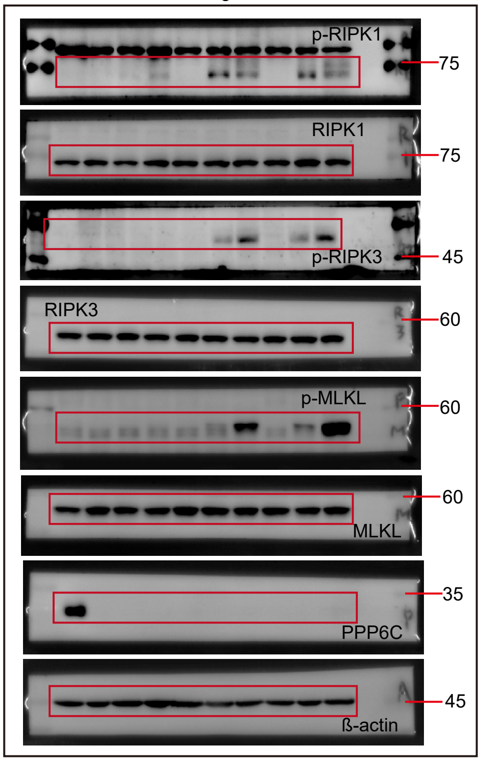

Uncropped blot images of the indicated Figures (page 5 of 5)

Fig.S5B

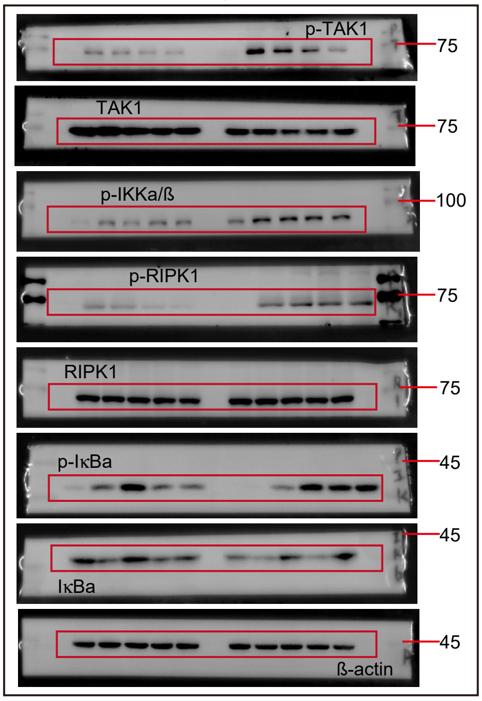

Fig. 6B

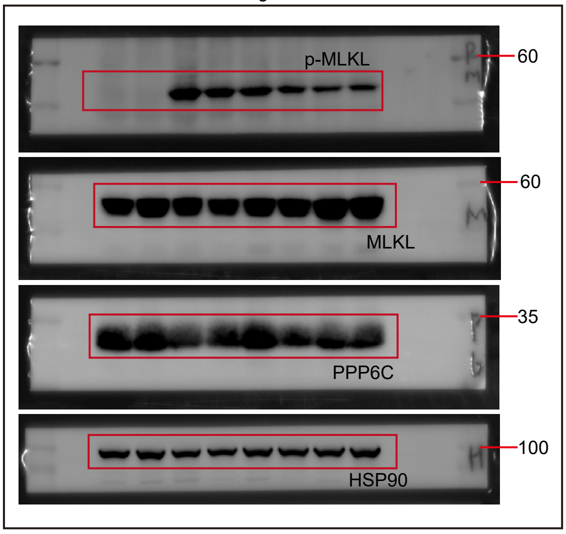

Fig. S6H

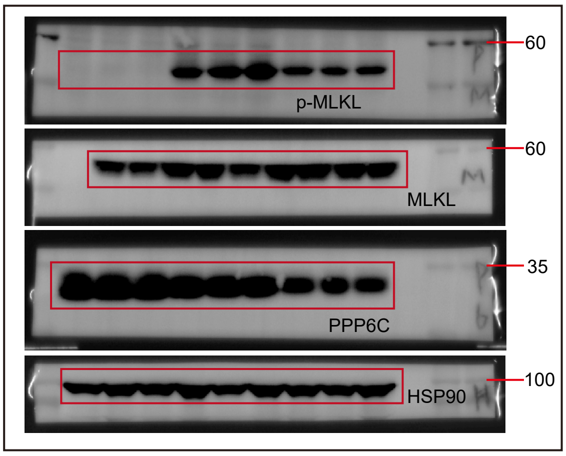

Supplement: Supplementary file 2 — Uncropped blot [file 41419_2022_5076_MOESM2_ESM.pdf]
