# Supplementary material for: Deficiency of PPP6C protects TNF-induced necroptosis through activation of TAK1
Source: Cell Death Dis. 2022 Jul 16;13(7):618. doi: 10.1038/s41419-022-05076-1 (PMC9288536; doi:10.1038/s41419-022-05076-1)
Supplement: Supplementary file 3 — supplementary figure legend and tables CDDIS-22-0857R2 [file 41419_2022_5076_MOESM3_ESM.docx]

**Supplementary figure legend**

**Figure S1.** **Deletion of PPP6C prevents TNF-induced necroptosis.**

(A) Histogram shows the rank order of the enriched sgRNAs targeting indicated genes.

(B) Proliferation rate of WT and *Ppp6c*-KO L929 cells was analyzed using CCK8 assay.

(C and D) *PPP6C*-knockdown HT29 cells were treated with TSZ for 6 h, then stained with PI and analyzed under a microscope (C) or quantified by flow cytometer (D). Scale bar, 50 μm.

(E) *PPP6C*-knockdown HT29 cells were treated with TSZ for the indicated time and the cell viability was measured by CCK8 assay.

(F) *PPP6C*-knockdown HT29 cells were treated with TSZ for the indicated time and cell lysates were resolved on non-reducing PAGE for immunoblot.

(G) Proliferation rate of WT and *Ppp6c*-KO MEF cells was measured using CCK8.

(H) Control and *Ppp6c*-KO L929 cells were treated with normal concentration (10 ng/ml TNF+20 μM Z-VAD-FMK, TZ-nc) or a lower concentration (100 pg/ml TNF+20 μM Z-VAD-FMK, TZ-lc) of TNF for the indicated time and quantified by flow cytometer after PI staining.

Data shown are representative of three independent experiments and presented as means ± SDs of triplicates (D, E, H) or six technical repeats (B, G). **p < 0.01, ***p < 0.001, with an unpaired Student’s t-test (B, G, H) or one-way ANOVA analysis (D, E).

**Figure S2. *Ppp6c* depletion reduces the expression RIPK3 and MLKL.**

(A) Immunoblot analysis of the endogenous protein levels in L929 cells as indicated.

(B) Schematic diagram of PPP6C phosphatase dead (PD) mutant.

(C) Immunoblot analysis of *PPP6C* knockdown HT29 cells with indicated antibodies.

(D) Quantitative RT-PCR analysis of mRNA expression level of necroptotic cell death related molecules in *PPP6C* knockdown HT29 cells.

(E) Quantitative RT-PCR analysis of mRNA expression level of necroptotic cell death related molecules in *Ppp6c*-KO or PPP6C re-expression L929 cells.

Data shown are representative of three independent experiments and presented as means ± SDs of three technical repeats (D, E). *p < 0.05, ***p < 0.001, with one-way ANOVA analysis (D, E).

**Figure S3. PPP6C represses TNF-induced NF-κB activity.**

(A and B) Control and *Ppp6c*-KO L929 cells were treated with TNF (10 ng/ml) for the indicated time. P65 localization was analyzed by immunofluorescence (A) and quantified in three independent experiments performed in duplicate (B). Scale bar, 50 μm.

(C) Control and *Ppp6c*-KO L929 cells were treated with TNF (10 ng/ml) for the indicated time. Cell lysates were probed with indicated antibodies.

(D) Control and *Ppp6c*-KO L929 cells were treated with TNF (10 ng/ml) for the indicated time. The relative mRNA expression of NF-κB target genes was quantified by quantitative RT-PCR analysis.

(E) HEK293T cells were transfected with NF-κB luciferase reporter plasmids together with indicated plasmids or treatment. Cell lysates were collected for luciferase assay 36 h after transfection.

(F) Control and *Ppp6c*-KO L929 cells were pretreated with P65 inhibitor (Maslinic acid, 20 μM) or IκBα inhibitor (BAY117085, 10 μM) for 30 min and then treated with TZ for 4 h. Cell lysates were probed with indicated antibodies.

(G) Control and *Ppp6c*-KO L929 cells were pretreated with TAK1 inhibitor (5Z-7-Oxozeaenol, 1 μM) or IKKα/β inhibitor (IKK16, 1 μM) for 30 min and then treated with TZ for 4 h. Cell lysates were probed with indicated antibodies.

(H) Control and *Ppp6c*-KO L929 cells were treated with TNF (10 ng/ml) for the indicated time, and then lysed for immunoblot with indicated antibodies.

Data shown are representative of three independent experiments and presented as means ± SDs of three technical repeats (B, D, E). **p < 0.01, ***p < 0.001, with an unpaired Student’s t-test (B, D) or one-way ANOVA analysis (E).

**Figure S4. Inhibition of P65 or IκBα does not affect RIPK1 phosphorylation at S166.**

(A and B) Control and *Ppp6c*-KO L929 cells were pretreated with P65 inhibitor (Maslinic acid, 20 μM) (A) or IκBα inhibitor (BAY117085, 10 μM) (B) for 30 min and then treated with TNF (10 ng/ml) for the indicated time. Cells were lysed for immunoblot with indicated antibodies.

(C) *TAK1*-KO HEK293T cells were transfected with IKKα/β and RIPK1 plasmids together with PPP6C plasmids for 36 h. Cell lysates were probed with indicated antibodies.

**Figure S5. TAK1 and IKKα/β were sustainably activated in *Tab2*-depletion L929 cells.**

(A) The knockdown efficiency of *Tab2* in control and *Ppp6c*-KO L929 cells were determined by quantitative RT-PCR.

(B) Control and *Tab2* knockdown L929 cells were treated with TNF (10 ng/ml) for the indicated time and then lysed for immunoblot with indicated antibodies.

(C) *Tab2*-knockdown L929 cells and *Tab2*-knockdown *Ppp6c*-KO L929 cells were pretreated with Necrostatin-1 (10 μM) for 30 min, and then treated with TZ for 8 h. Cells were stained with PI and quantified by flow cytometer.

Data shown are representative of three independent experiments and presented as means ± SDs of three technical repeats (A, C). **p < 0.01, ***p < 0.001, with one-way ANOVA analysis (A, C).

**Figure S6. *Ppp6c* loss alleviates DSS-induced colitis in colon epithelium.**

(A) Cryosections of colon tissues from *Rosa26^tdTomato/+^* and *Shh^Cre/+^*;*Rosa26^tdTomato/+^* mice were stained with anti-E-cadherin antibody and evaluated under fluorescent microscope. Scale bar, 100 μm.

(B and C) Colon length of *Ppp6c*^f/+^ (n=3) and *Shh^Cre/+^*; *Ppp6c*^f/+^ (n=3) mice without any treatment.

(D) Body weights of *Ppp6c*^f/+^ (n=3) and *Shh^Cre/+^*;*Ppp6c*^f/+^ (n=3) mice were determined every day during the DSS treatment as indicated.

(E) Gross morphology of the colons from DSS-treated *Ppp6c*^f/+^ (n=3) and *Shh^Cre/+^*;*Ppp6c*^f/+^ (n=3) mice.

(F) Colon length of *Ppp6c*^f/+^ (n=3) and *Shh^Cre/+^*;*Ppp6c*^f/+^ (n=3) mice was measured after DSS treatment.

(G) Representative H&E staining of colons from DSS-treated *Ppp6c*^f/+^ and *Shh^Cre/+^*;*Ppp6c*^f/+^ mice. Scale bar, 100 μm.

(H) Colonic proteins from DSS-treated *Ppp6c*^f/+^ and *Shh^Cre/+^*;*Ppp6c*^f/+^ mice were analyzed by immunoblot with indicated antibodies.

(I) TUNEL staining on colon sections from DSS-treated *Ppp6c*^f/+^ and *Shh^Cre/+^*; *Ppp6c*^f/+^ mice. Scale bar, 100 μm.

Data are presented as mean ± SD (C, D, F). *p < 0.05, **p < 0.01, ***p < 0.001, with an unpaired Student’s t-test (C, D) or one-way ANOVA analysis (F).

**Table S1. Sequences for sgRNA, shRNA and qRT-PCR primers.**

| sgRNA mouse *Ppp6c* KO | 5'-TGTGAGCATATCAAAAACTT-3' |
| --- | --- |
| sgRNA human TAK1 KO | 5'-GAGTTGTTTGCAAAGCTAAG-3' |
| shRNA human PPP6C #1 | 5'-GAGTCAAATGTTCAGCCAGTA-3' |
| shRNA human PPP6C #2 | 5'-GCTTCGATCATGGTCTTCAAA-3' |
| shRNA mouse *Tab2* #1 | 5'-CCTCGAAAGAACCAGATAGAA-3' |
| shRNA mouse *Tab2* #2 | 5'-AGTCAACCCAAGGTCTATATT-3' |
| mouse *A20* forward | 5'-CTCAGAACCAGAGATTCCATGAAG-3' |
| mouse *A20* reverse | 5'-CCTGTGTAGTTCGAGGCATGT-3' |
| mouse *Tnf* forward | 5'-AGCCGATGGGTTGTACCTTG-3' |
| mouse *Tnf* reverse | 5'-ATAGCAAATCGGCTGACGGT-3' |
| mouse *Iκb* forward | 5'-GATCCGCCAGGTGAAGGG-3' |
| mouse *Iκb* reverse | 5'-GCAATTTCTGGCTGGTTGG-3' |
| mouse *Ciap1* forward | 5'-CGCAGCCCGTATTAGAAC-3' |
| mouse *Ciap1* reverse | 5'-AGATTCCCAGCACCTCAG-3' |
| mouse *Tab2* forward | 5'-CATGACCTGCGACAAAAATTCC-3' |
| mouse *Tab2* reverse | 5'-TGATTGCGTAGACCAGAAATTCC-3' |

**Table S2. Antibodies and reagents.**

| Antibodies | | | | | |
| --- | --- | --- | --- | --- | --- |
| REAGENT or RESOURCE | SOURCE | | IDENTIFIER | | DILUTION |
| Anti-p-RIPK1(S166） | Cell Signaling | | #53286 | | 1:800(WB) |
| Anti-p-RIPK1(S166） | Cell Signaling | | #44590 | | 1:800(WB) |
| Anti-RIPK1 | BD Biosciences | | 610458 | | 1:1000(WB) |
| Anti-p-RIPK3(T231/S232) | Abcam | | ab222320 | | 1:1000(WB) |
| Anti-RIPK3 | Novus Biologicals | | NBP1-77299SS | | 1:1000(WB) |
| Anti-p-MLKL(S345) | Abcam | | ab196436 | | 1:1000(WB) |
| Anti-p-MLKL(S358) | Abcam | | ab187091 | | 1:1000(WB) |
| Anti-MLKL | Abcam | | ab243142 | | 1:1000(WB) |
| Anti-HSP90 | Abcam | | ab203085 | | 1:5000(WB) |
| Anti-β-actin | Abclonal | | AC026 | | 1:5000(WB) |
| Anti-PPP6C | Abcam | | ab131335 | | 1:1000(WB) |
| Anti-p-IKKα/β | Cell Signaling | | #2697 | | 1:800(WB) |
| Anti-p-IκBα | Cell Signaling | | #2859 | | 1:1000(WB) |
| Anti-IκBα | Cell Signaling | | #9242 | | 1:1000(WB) |
| Anti-p-P65 | Cell Signaling | | #3033 | | 1:1000(WB) |
| Anti-P65 | Cell Signaling | | #6956 | | 1:1000(WB)  1:500(IF) |
| Anti-p-P38 | Cell Signaling | | #9215 | | 1:1000(WB) |
| Anti-P38 | Cell Signaling | | #9212 | | 1:1000(WB) |
| Anti-p-Erk1/2 | Cell Signaling | | #9101 | | 1:1000(WB) |
| Anti-Erk1/2 | Cell Signaling | | #4695 | | 1:1000(WB) |
| Anti-P-TAK1(T187) | ImmunoWay | | YP0424 | | 1:800(WB) |
| Anti-P-TAK1(T187) | Cell Signaling | | #4536 | | 1:600(WB) |
| Anti-TAK1 | Abcam | | ab109526 | | 1:1000(WB) |
| E-Cadherin | Cell Signaling | | #14472 | | 1:500(IF) |
| Anti-FLAG | Sigma | | F1804 | | 1:2000(WB) |
| Anti-HA | Roche | | 11867423001 | | 1:2000(WB) |
| Anti-HA Magnetic Beads | Thermo | | 88836 | | 1:200(IP) |
| Anti-FLAG Magnetic Beads | Sigma | | M8823 | | 1:200(IP) |
| Chemicals and Kits | | | | | |
| REAGENT or RESOURCE | | SOURCE | | IDENTIFIER | |
| mTNFα | | novoprotein | | CF09 | |
| hTNFα | | novoprotein | | C008 | |
| Z-VAD-FMK | | MedChemExpress | | HY-16658B | |
| Apoptosis Inducer Kit | | Beyotime | | C0006S | |
| Propidium Iodide (PI) | | Beyotime | | ST511 | |
| DAPI | | Sigma | | 28718-90-3 | |
| 5Z-7-Oxozeaenol | | MedChemExpress | | HY-12686 | |
| IKK 16 | | MedChemExpress | | HY-13687 | |
| Adezmapimod | | MedChemExpress | | HY-10256 | |
| MK2-IN-1 | | MedChemExpress | | HY-12834 | |
| BAY 11-7085 | | MedChemExpress | | HY-10257 | |
| Maslinic acid | | MedChemExpress | | HY-N0629 | |
| Necrostatin-1 | | MedChemExpress | | HY-15760 | |
| Cell Counting Kit-8 | | Beyotime | | C0038 | |
| TUNEL Apoptosis Detection Kit | | YEASEN | | 40307ES20 | |
| Dextran sulfate sodium | | Bidepharm | | BD123894 | |

**Table S3. All screen hits with sgRNA enrichment (p＜0.05).**

| **Gene name** | **log2 sgRNA (fold change)** | **p-Value** | **Rank** |
| --- | --- | --- | --- |
| *Tnfrsf1a* | 11.059 | 2.23E-07 | 1 |
| *Ripk3* | 9.4397 | 2.23E-07 | 2 |
| *Ripk1* | 9.2543 | 2.23E-07 | 3 |
| *Mlkl* | 6.5036 | 2.23E-07 | 4 |
| *Cyld* | 5.9201 | 2.23E-07 | 5 |
| *Nfkbia* | 2.5185 | 2.90E-06 | 6 |
| *Tradd* | 2.3708 | 6.92E-06 | 7 |
| *mmu-mir-669b* | 1.5592 | 2.08E-05 | 8 |
| *Gm20816* | 1.4765 | 2.74E-05 | 9 |
| *Ppp6c* | 1.3984 | 4.32E-04 | 10 |
| *mmu-mir-467a-3* | 1.3136 | 4.32E-04 | 11 |
| *Olfr1506* | 1.3136 | 4.51E-04 | 12 |
| *Spata2* | 1.2787 | 5.16E-04 | 13 |
| *Dmrtc1c2* | 1.22 | 6.30E-04 | 14 |
| *Fosl1* | 1.1039 | 6.65E-04 | 15 |
| *Gm13277* | 1.1012 | 6.65E-04 | 16 |
| *Pfdn5* | 1.0953 | 6.81E-04 | 17 |
| *Shoc2* | 1.0829 | 7.71E-04 | 18 |
| *Zfp68* | 1.0465 | 1.03E-03 | 19 |
| *Galntl5* | 0.98918 | 1.03E-03 | 20 |
| *Zfp217* | 0.87712 | 1.42E-03 | 21 |
| *Socs3* | 0.87712 | 1.49E-03 | 22 |
| *H2-Q9* | 0.87712 | 1.56E-03 | 23 |
| *Vmn1r37* | 0.87712 | 1.56E-03 | 24 |
| *Pcdhga1* | 0.86965 | 1.56E-03 | 25 |
| *Pcdhgb2* | 0.81344 | 1.62E-03 | 26 |
| *Cd79a* | 0.78096 | 1.74E-03 | 27 |
| *2310011J03Rik* | 0.78096 | 1.74E-03 | 28 |
| *mmu-mir-6378* | 0.78096 | 1.74E-03 | 29 |
| *Mrgpra2b* | 0.78096 | 1.74E-03 | 30 |
| *Pcdhgb5* | 0.76321 | 2.26E-03 | 31 |
| *Gm20806* | 0.71571 | 2.48E-03 | 32 |
| *Xlr5b* | 0.64009 | 2.48E-03 | 33 |
| *Foxp1* | 0.62405 | 2.60E-03 | 34 |
| *Zfp534* | 0.61164 | 3.09E-03 | 35 |
| *Actg1* | 0.60365 | 5.54E-03 | 36 |
| *Itga5* | 0.56274 | 7.15E-03 | 37 |
| *Pcbp1* | 0.56274 | 7.15E-03 | 38 |
| *Tnfrsf11a* | 0.56274 | 7.15E-03 | 39 |
| *Elovl5* | 0.56274 | 7.15E-03 | 40 |
| *Lhpp* | 0.56274 | 8.26E-03 | 41 |
| *Ggnbp2* | 0.54278 | 9.45E-03 | 42 |
| *Hp1bp3* | 0.53705 | 1.18E-02 | 43 |
| *mmu-mir-292* | 0.53705 | 1.18E-02 | 44 |
| *Wdr86* | 0.51401 | 1.24E-02 | 45 |
| *Gpc3* | 0.51344 | 1.30E-02 | 46 |
| *Pusl1* | 0.50749 | 1.63E-02 | 47 |
| *Mtf1* | 0.47698 | 1.78E-02 | 48 |
| *0610040J01Rik* | 0.47698 | 1.78E-02 | 49 |
| *Prkaca* | 0.45578 | 2.22E-02 | 50 |
| *Gm4498* | 0.45346 | 2.22E-02 | 51 |
| *Gm4175* | 0.41173 | 2.61E-02 | 52 |
| *Alg8* | 0.40583 | 3.07E-02 | 53 |
| *Park7* | 0.40583 | 3.07E-02 | 54 |
| *Rnaset2b* | 0.39543 | 3.09E-02 | 55 |
| *Ddt* | 0.37829 | 3.29E-02 | 56 |
| *mmu-mir-6342* | 0.37829 | 3.29E-02 | 57 |
| *Npc1l1* | 0.35058 | 3.55E-02 | 58 |
| *mmu-mir-297a-4* | 0.33923 | 3.63E-02 | 59 |
| *mmu-mir-466a* | 0.33018 | 3.63E-02 | 60 |
| *mmu-mir-294* | 0.30967 | 3.63E-02 | 61 |
| *Jak3* | 0.28927 | 3.63E-02 | 62 |
| *St18* | 0.28927 | 3.74E-02 | 63 |
| *Flrt3* | 0.28927 | 3.74E-02 | 64 |
| *Gp5* | 0.28927 | 3.74E-02 | 65 |
| *Ypel5* | 0.28927 | 3.74E-02 | 66 |
| *Commd10* | 0.28927 | 3.78E-02 | 67 |
| *Naip7* | 0.28927 | 3.78E-02 | 68 |
| *Tgtp2* | 0.278 | 4.14E-02 | 69 |
| *Zeb2* | 0.26444 | 4.15E-02 | 70 |
| *Gbp8* | 0.26444 | 4.22E-02 | 71 |
| *Try10* | 0.24836 | 4.55E-02 | 72 |
| *Prss57* | 0.24836 | 4.55E-02 | 73 |
| *Fndc3b* | 0.24836 | 4.56E-02 | 74 |
| *Rerg* | 0.24836 | 4.67E-02 | 75 |
| *Hivep2* | 0.24836 | 4.72E-02 | 76 |
| *Mroh7* | 0.24836 | 4.72E-02 | 77 |
| *Lce1j* | 0.24836 | 4.78E-02 | 78 |
| *Nprl3* | 0.24836 | 4.94E-02 | 79 |
