# Supplementary figures and images for: Deficiency of PPP6C protects TNF-induced necroptosis through activation of TAK1
Source: Cell Death Dis. 2022 Jul 16;13(7):618. doi: 10.1038/s41419-022-05076-1 (PMC9288536; doi:10.1038/s41419-022-05076-1)

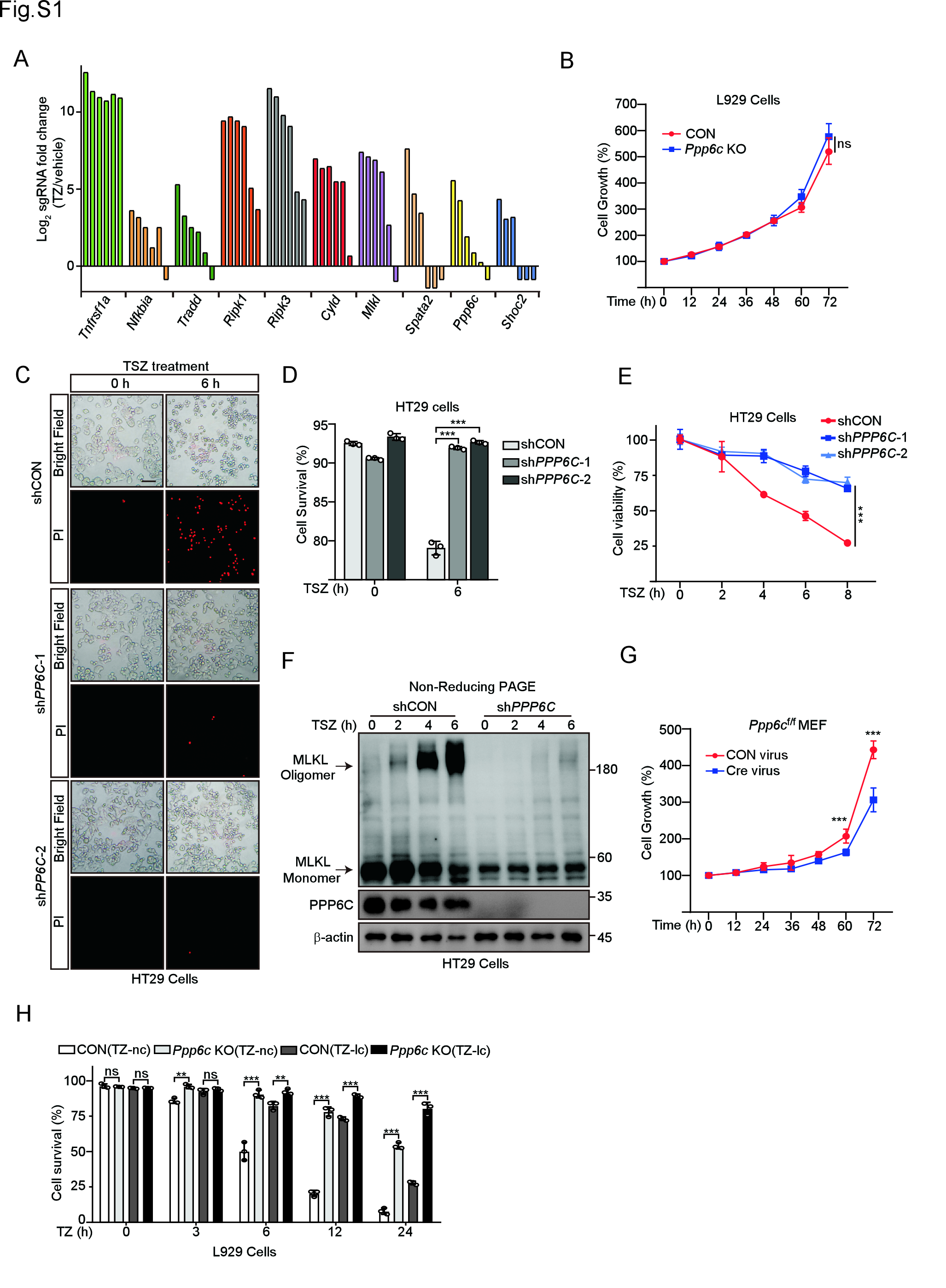

Supplement: Supplementary file 4 — Figure S1 [file 41419_2022_5076_MOESM4_ESM.tif]

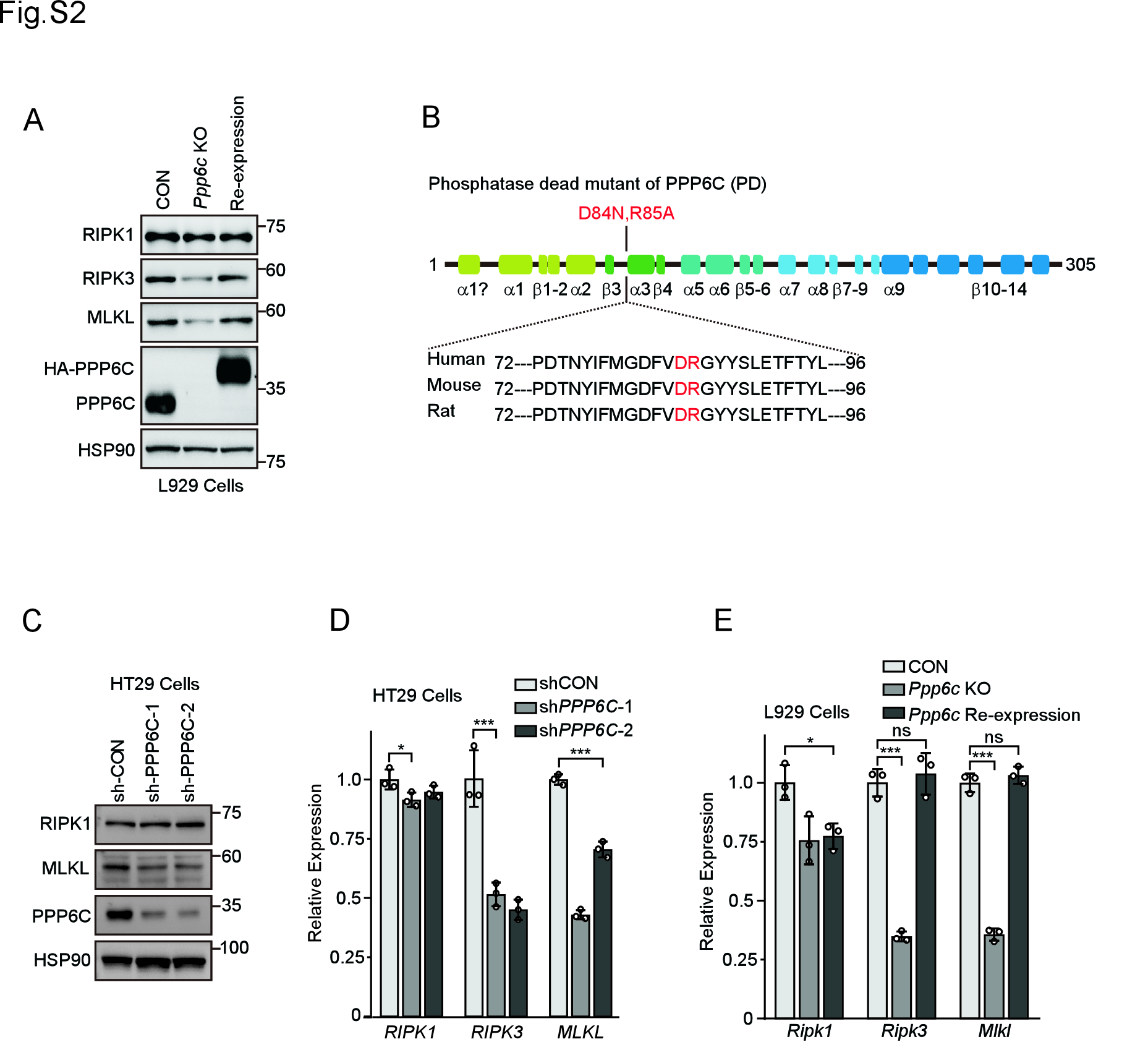

Supplement: Supplementary file 5 — Figure S2 [file 41419_2022_5076_MOESM5_ESM.tif]

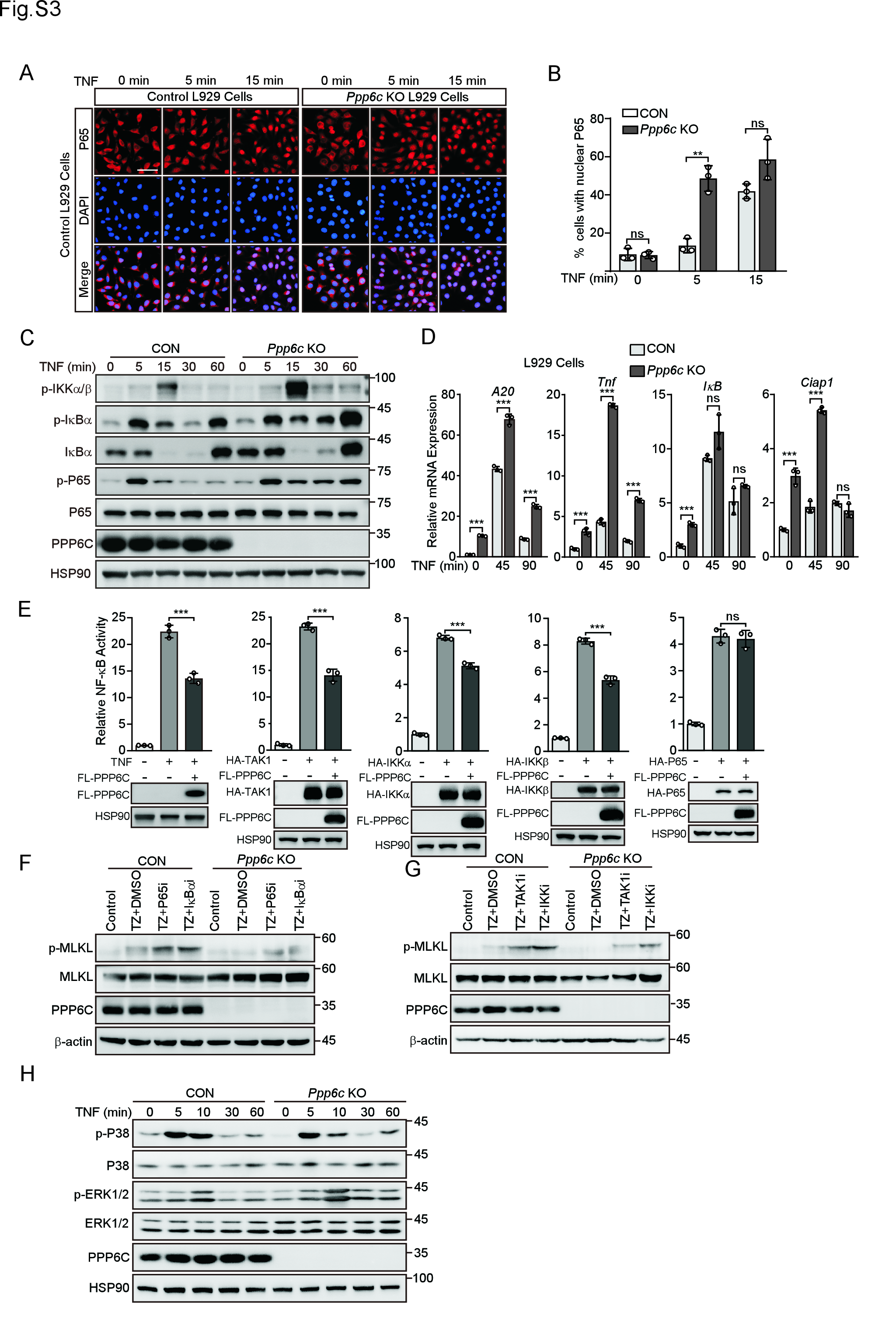

Supplement: Supplementary file 6 — Figure S3 [file 41419_2022_5076_MOESM6_ESM.tif]

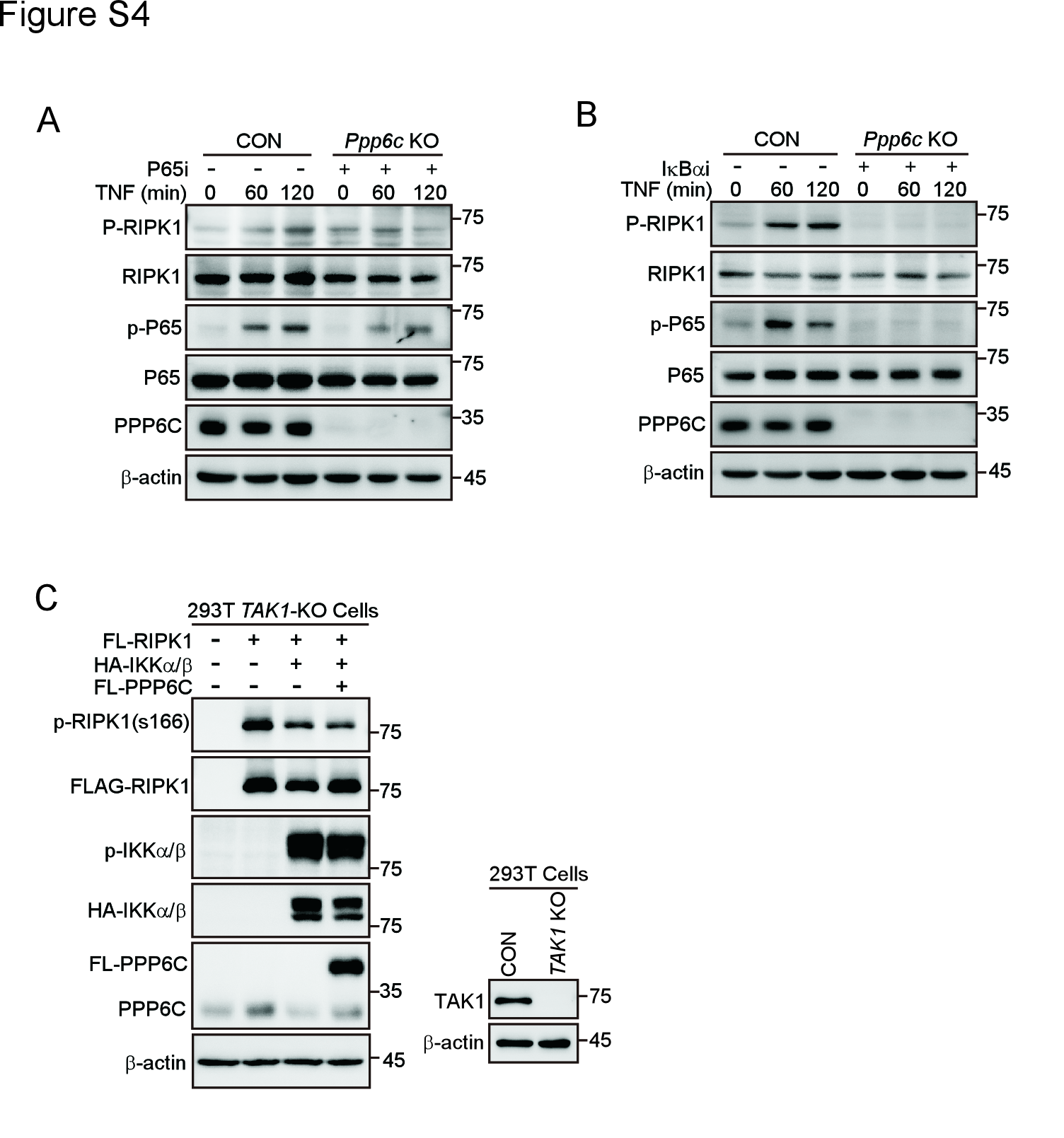

Supplement: Supplementary file 7 — Figure S4 [file 41419_2022_5076_MOESM7_ESM.tif]

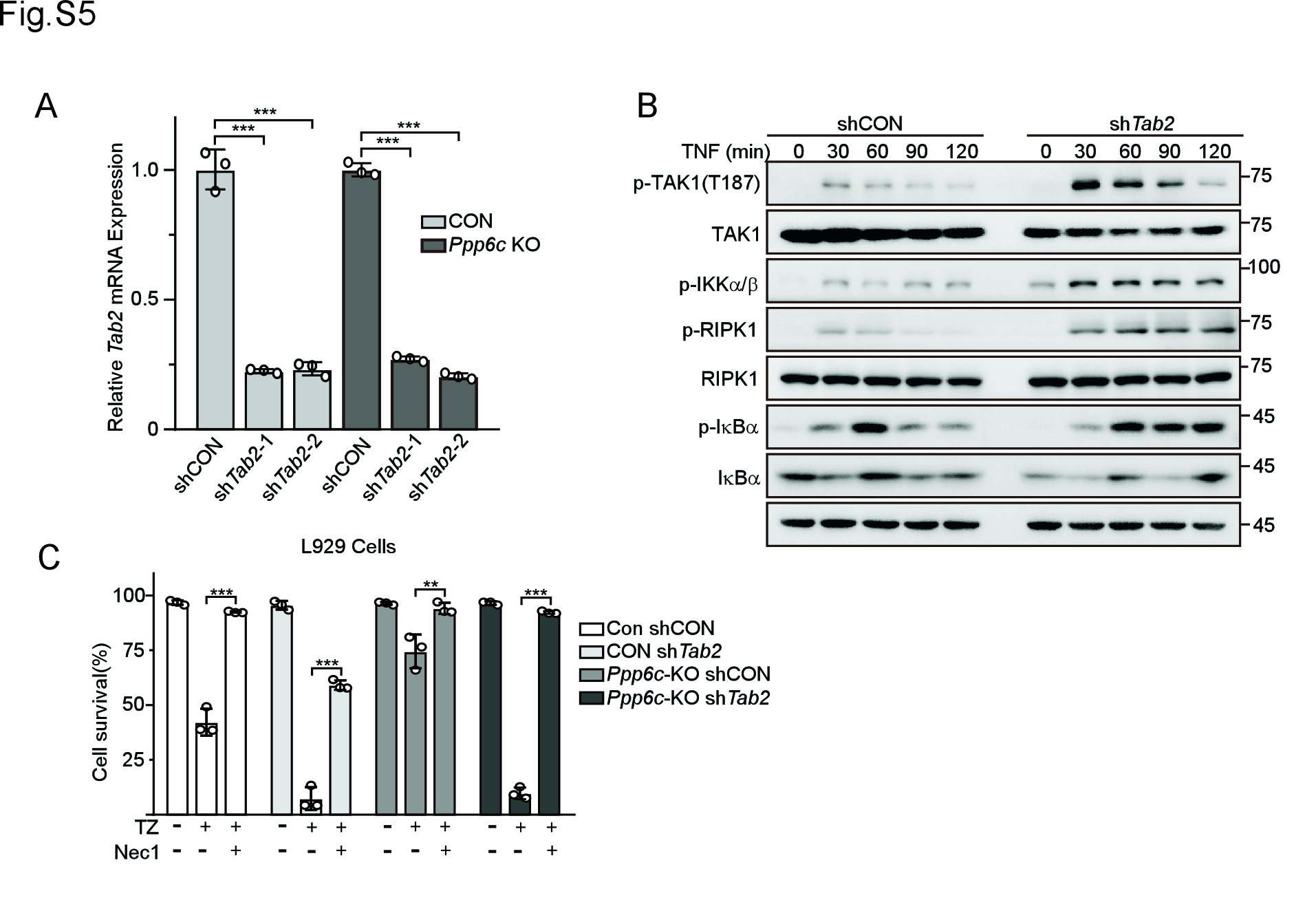

Supplement: Supplementary file 8 — Figure S5 [file 41419_2022_5076_MOESM8_ESM.tif]

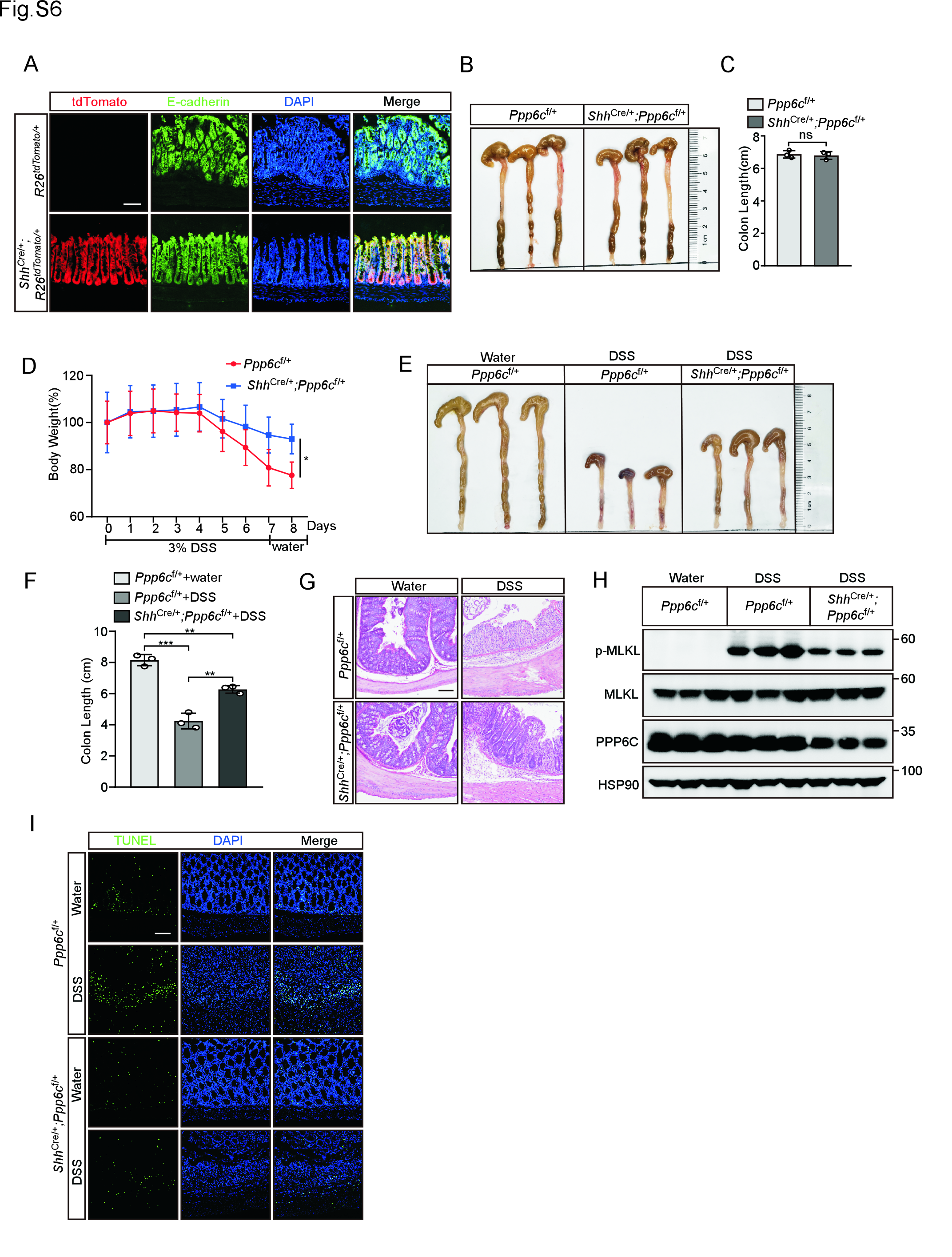

Supplement: Supplementary file 9 — Figure S6 [file 41419_2022_5076_MOESM9_ESM.tif]
